# The proteomic profile is altered but not repaired after bariatric surgery in type 2 diabetes pigs

**DOI:** 10.1038/s41598-024-60022-9

**Published:** 2024-05-03

**Authors:** Karolina Ferenc, Michał Marcinkowski, Jarosław Olszewski, Paweł Kowalczyk, Tomaš Pilžys, Damian Garbicz, Naser Dib, Bianka Świderska, Piotr Matyba, Zdzisław Gajewski, Elżbieta Grzesiuk, Romuald Zabielski

**Affiliations:** 1 Center for Translational Medicine, Warsaw University of Life Sciences, Nowoursynowska 100, 02-797 Warsaw, Poland; 2 Institute of Genetics and Biotechnology, Faculty of Biology, University of Warsaw, Pawińskiego 5a, 02-106 Warsaw, Poland; 3 Institute of Biochemistry and Biophysics, Polish Academy of Sciences, Pawińskiego 5a, 02-106 Warsaw, Poland; 4 Kielanowski Institute of Animal Physiology and Nutrition, Polish Academy of Sciences, Instytucka 3, 05-110 Jabłonna, Poland; 5 Institute of Oncology, Maria Sklodowska-Curie National Research, W.K. Roentgena 5, 02-781 Warsaw, Poland; 6 European Health Centre Otwock (ECZ Otwock), The Fryderyk Chopin Hospital, Borowa 14/18, 05-400 Otwock, Poland

**Keywords:** Pig model, Diabetes, High energy diet, Scopinaro bariatric surgery, Proteomic studies, DNA modifications, ALKBH proteins, Type 2 diabetes, Animal physiology

## Abstract

To reveal the sources of obesity and type 2 diabetes (T2D) in humans, animal models, mainly rodents, have been used. Here, we propose a pig model of T2D. Weaned piglets were fed high fat/high sugar diet suppling 150% of metabolizable energy. Measurements of weight gain, blood morphology, glucose plasma levels, cholesterol, and triglycerides, as well as glucose tolerance (oral glucose tolerance test, OGTT) were employed to observe T2D development. The histology and mass spectrometry analyses were made *post mortem*. Within 6 months, the high fat-high sugar (HFHS) fed pigs showed gradual and significant increase in plasma triglycerides and glucose levels in comparison to the controls. Using OGTT test, we found stable glucose intolerance in 10 out of 14 HFHS pigs. Mass spectrometry analysis indicated significant changes in 330 proteins in the intestine, liver, and pancreas of the HFHS pigs. These pigs showed also an increase in DNA base modifications and elevated level of the ALKBH proteins in the tissues. Six diabetic HFHS pigs underwent Scopinaro bariatric surgery restoring glycaemia one month after surgery. In conclusion, a high energy diet applied to piglets resulted in the development of hyperlipidaemia, hyperglycaemia, and type 2 diabetes being reversed by a bariatric procedure, excluding the proteomic profile utill one month after the surgery.

## Introduction

Insulin resistance is a complex metabolic disorder. It can be of genetic or environmental etiology^1^. Following the generally accepted theories Murri et al.^2^ demonstrated that the pathogenesis of insulin resistance-related obesity may be associated with chronic low level inflammation present in adipose tissue and changes in adipokine secretion. Also oxidative stress appears to be a deleterious factor leading to insulin resistance, dyslipidaemia, β-cell dysfunction, impaired glucose tolerance, and ultimately, to type 2 diabetes (T2D)^3^. Chronic oxidative stress, hyperglycaemia, and dyslipidaemia are particularly dangerous to β-cells, which under conditions of lowest antioxidant levels show high requirements for oxidative energy, leading to DNA damage, decrease in the expression of key β-cell genes, and induction of cell death^4^. Impairment of the β-cell functions results in underproduction of insulin, disturbed glucose-stimulated insulin secretion, fasting hyperglycaemia, and eventually leads to the development of T2D. Recently, we have found that the FTO dioxygenase, one out of the nine human ALKBH family proteins, is located exclusively in pancreatic β-cells, implicating its involvement in T2D^5^. Bariatric surgeries, originally intended to treat morbidly obese patients with body mass index (BMI) of ca. 40, proved to be an effective treatment against T2D^6^. The effectiveness of bariatric surgery depends on the type of the surgical procedure and ranges from 43 to 98%^7^. Procedures with malabsorptive components, such as Roux-en-Y gastric bypass (RYGB) and biliopancreatic diversion with duodenal switch (BPD-DS) are considered effective. However, the bilio-pancreatic diversion (BPD) and the bariatric procedure with creation of gastric sleeve (Scopinaro method) is considered the gold standard with the highest efficacy for the T2D patient^8^. It has been found that bariatric procedures diminish insulin resistance and T2D manifestation just a few days after the surgery, whereas the reduction of body mass proceeds gradually for several months, indicating the involvement of distinct mechanisms^9^. Using a rat model, we have demonstrated that the relief of T2D symptoms is weight loss-independent and involves neurohormonal pathways^10^.

Rodents are the most commonly used model to study the development and pathogenesis of obesity and T2D. However, the T2D rodents show several genetic defects interfering with the desirable results^11^. Moreover, the rodent model shows large differences when compared to human pathophysiology, leading to difficulties in translating animal results into humans. For instance, studies on the ALKBH family proteins have shown that certain genetic modifications within the *Fto* gene encoding the FTO protein, the ninth ALKBH homolog, are lethal in humans, but not in mice^12^. The pig T2D model appears to be much more reliable in this respect^5^. Selection of pigs in T2D research is based on the results of drugs and devices tested in pigs showing high positive predictive value for subsequent human outcomes^13,14^ due to the high similarity in the pharmacokinetics following drug administration, gastrointestinal tract structure and function, pancreatic morphology, and overall metabolic pig status^15–17^. So far, several models of diabetic pigs have been developed. In some of these models the diabetes was induced in Yucatan, Sinclair, and Yorkshire pigs by damaging the pancreatic β-cells with streptozotocin or alloxan^14,18,19^. This type of pharmacologically induced model is useful for studying insulin-dependent diabetes type 1, rather than type 2. To track the subtle changes affecting the development and/or remission of insulin resistance, a better T2D model is needed, one that mimicks the pathophysiology processes in humans. As type 2 diabetes is most often associated with obesity, a good model of T2D should be based on dietary modifications leading to the development of T2D as a component of metabolic syndrome^14^.

The aim of this study was to develop and evaluate the type 2 diabetes pig model induced exclusively by dietary treatment with an excessive intake of disaccharides (sugar) and fatty acids (rapeseed oil) and to investigate its organs critical for the insulin resistance development at structural and molecular level. The estimation of the structure and function of the small intestine was supported by proteomic profiling of tissues, and evaluation of DNA base modifications. The investigation was further extended into proteomic studies of the tissues affected by the diet: liver, pancreas, and adipose tissue. The levels of the ALKBH proteins responsible for the dealkylation of the modified DNA bases were studied as well.

The second step, important for the future use of our model to study the pathomechanisms of T2D and to search for new drugs and methods improving insulin sensitivity is to reverse diabetes by the method of bariatric surgery. Selected diabetic pigs underwent the Scopinaro bariatric surgery. These animals were examined one month after the surgery to determine the improvement of their insulin sensitivity markers and the level of modifications in the protein profile of their tissues. Since the levels of the ALKBH 2, 3, 4 and 8 proteins were elevated in pigs fed high fat-high sugar (HFHS) diet, we tried to determine if these proteins, removing base modifications from DNA and RNA, participate in the development of type 2 diabetes.

## Results

The high energy diet given from 42 days of life led to dramatic reduction of skeletal muscles growth and increase of abdominal and subcutaneous fat as compared to pigs fed control diet (Table 1). A 6 months after the onset of HFHS diet the animals were much lighter as compared to the controls, less mobile with spiky hair, they also manifested polydipsia and polyuria. The latter two symptoms disappeared ca. 2 weeks after the bariatric surgery. Bariatric surgery led to reduction of the body weight in HFHS pigs.

**Table 1:** Body weighy of animals during experiment. Control diet (C, n = 6), high energy diet (HFHS, n = 6), and high energy diet followed by bariatric surgery (BAR, n = 6).

	C	HFHS	BAR	*p*-value
PD 0	1.48 ± 0.08	1.5 ± 0.8	1.46 ± 0.07	NS
PD 42	14.97 ± 0.89	14.52 ± 0.59	15.06 ± 0.64	NS
PD 270	200,5 ± 11.4^a^	103.5 ± 7.5^b^	107.5 ± 8.71^b^	< 0.0001
PD 300			84.8 ± 7.24^c^	< 0.0001*

Mean and SD, One-way ANOVA, Tukey’s post-test.

*paired test between BAR group before (PD270) and one month after (300 PD) bariatric surgery.

### Influence of high energy diet on the results of pig’s blood analysis

**Figure 1:**
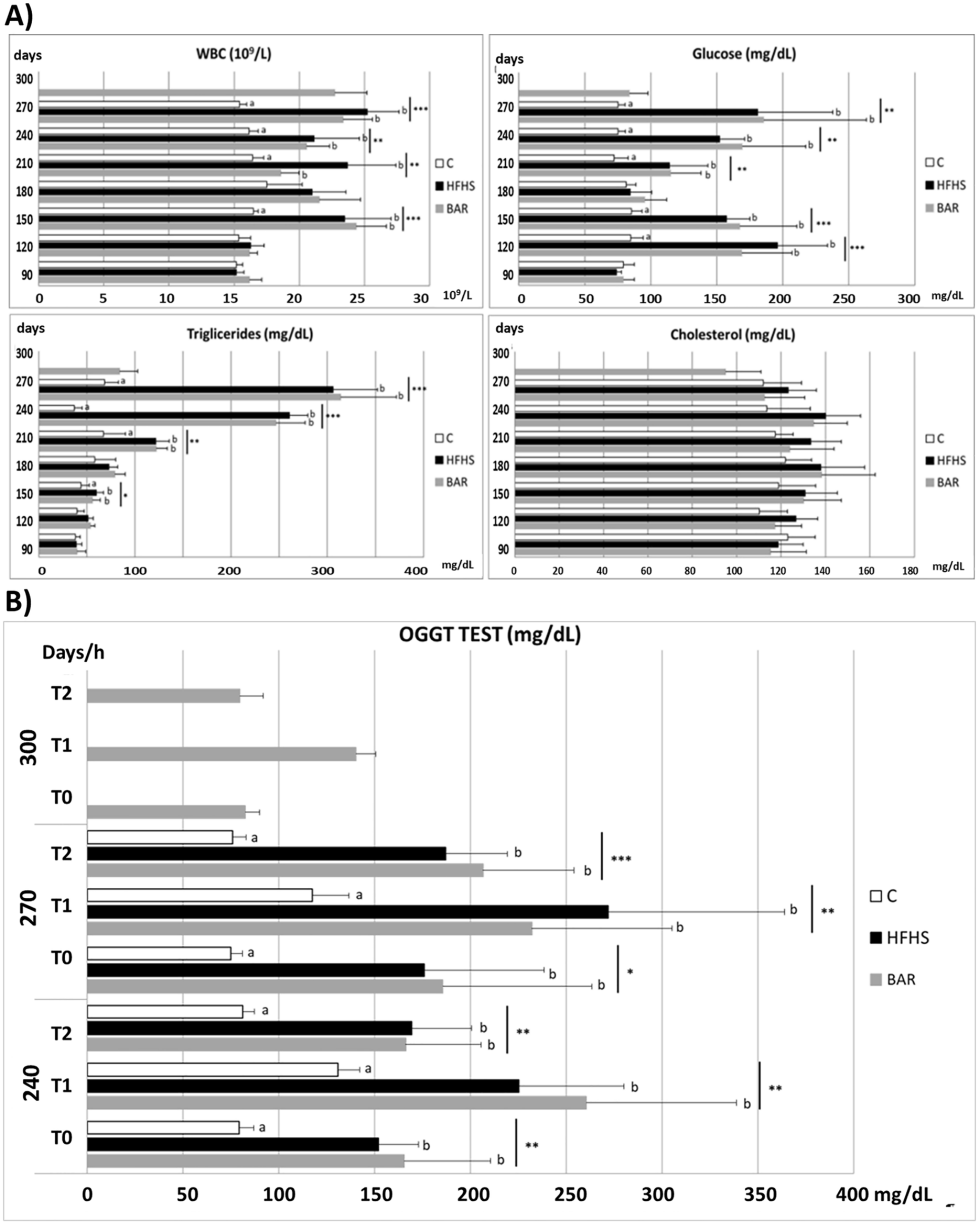
(**A**) Blood test results—Pigs on high energy versus normal diet, developing insulin resistance, and subjected to bariatric surgery. White blood cells count (WBC, × 10^9^/L, reference values: 10–22 × 10^9^/L), and concentrations of glucose (mg/dl, reference values: 65–95 mg/dL), triglycerides (mg/dl, reference values: 18–44 mg/dL) and cholesterol (mg/dl, reference values: 77–95 mg/dL) in pigs fed the control (C, n = 6) and high energy diet (HFHS, n = 14, and BAR, n = 6). Note, two bars represent the pigs fed a high energy diet: HFHS and BAR. The HFHS—represents all pigs that were fed HFHS diet, including animals that did not develop type 2 diabetes, and BAR—represents 6 out of 10 pigs that developed type 2 diabetes and were subjected to bariatric surgery 9 months after the onset of the HFHS diet (on PD270). The BAR pigs were switched from HFHS to the C diet after the bariatric surgery and were sacrificed one month after the bariatric surgery (on PD300). The asterisks indicate differences between the three (C, HFHS, and BAR) groups. Mean ± SD, one-way ANOVA and Tukey test. (**B**) Oral glucose tolerance test (mg/dl, pre-prandial reference values: 65–95 mg/dL) in pigs fed control diet (C, n = 6), high energy diet (HFHS, n = 14), and high energy feed, and pigs that underwent bariatric surgery (BAR, n = 6). The data shown on days PD240 and PD270 involve the C, HFHS and the BAR pigs; the data on day PD300 apply only to the HFHS pigs that underwent bariatric surgery (BAR). Blood sampling: T0, T1 and T2—just before and 1 and 2 h after glucose intake, respectively.

The high energy diet caused a biphasic elevation in the counts of white blood cells and glucose concentration (Fig. 1A). The first transient elevation appeared 2–3 months after the onset of the HFHS diet. The second, stable elevation was observed from the 6–7th month after the onset of the HFHS diet concomitantly with impaired glucose tolerance (Fig. 1B) and increase in the concentration of blood triglycerides. In contrast to glucose, the first elevation was not observed in blood triglycerides. One month after bariatric surgery, the plasma glucose and the triglycerides concentrations, but not the white blood cell counts, were normalized. The total cholesterol concentration was high in all pigs and did not show significant variations throughout the study (Fig. 1A), though, as mentioned earlier, only the pigs that naturally express high cholesterol in the blood were selected for the present study. The results of the oral glucose tolerance test (Fig. 1B) showed significant increase in glucose concentrations, both, before and after the glucose load, in the HFHS and BAR groups in comparison to the control pigs (C). In pigs fed high fat and high sugar diet, the tolerance to oral glucose was regained one month after the bariatric surgery (Fig. 1B).

### The influence of high energy diet on the pig’s tissue morphology

**Figure 2:**
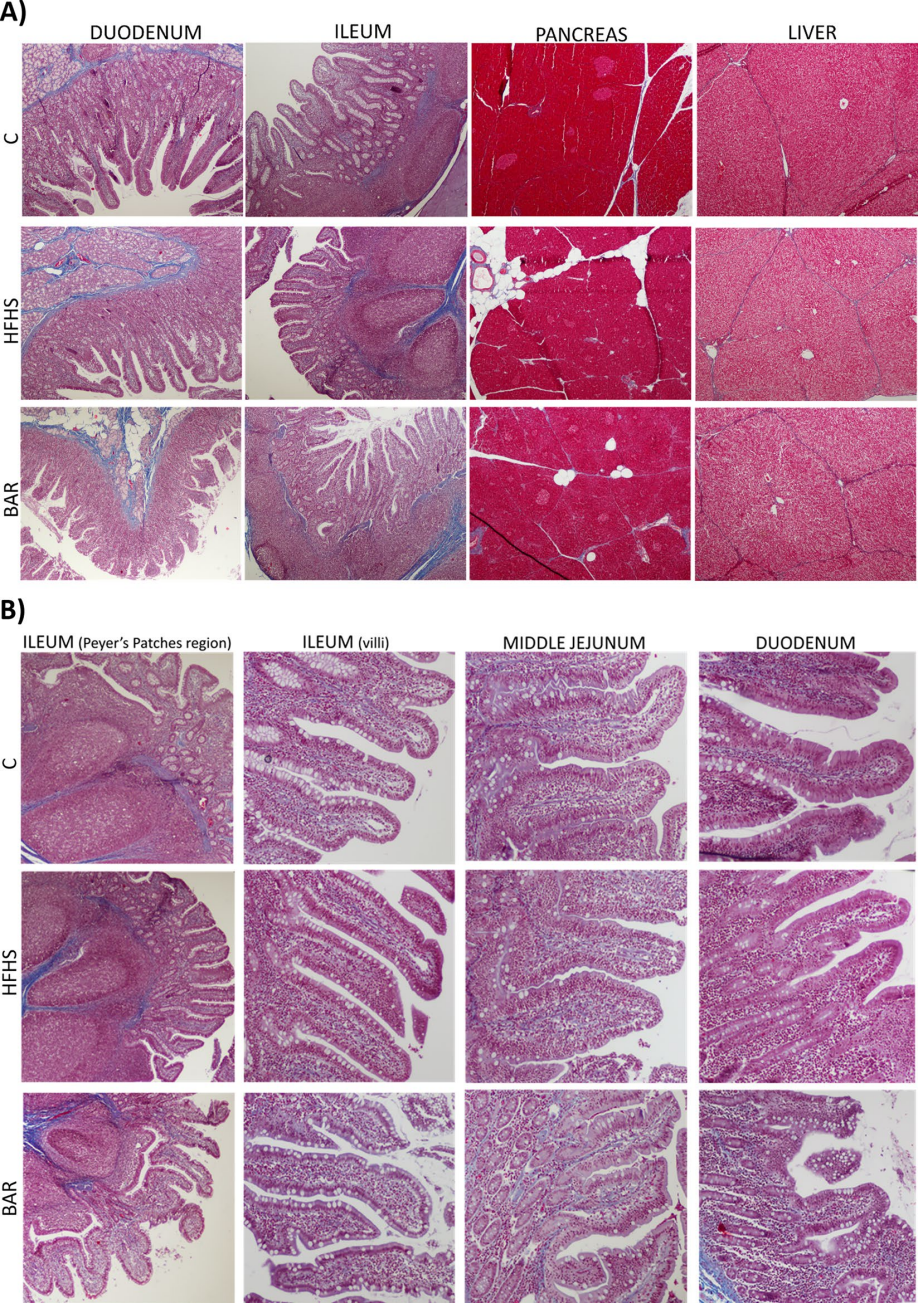
(**A**) Ultrastructure of the small intestine (duodenum and ileum), pancreas, and liver in pigs fed control diet (C), high energy diet (HFHS), and one month following bariatric surgery (BAR)—representative images. Haematoxylin and eosin staining, obj × 4. (**B**) Immunological barrier elements in the area of ileal Peyer’s patches region (left), ileal villi, jejunal and duodenal (right), in C, insulin resistant (HFHS), and insulin resistant pigs one month after the bariatric surgery (BAR). Trichrome staining, objective 10×. Note: in the BAR pigs, the duodenum corresponds to the enzymatic loop, the distal jejunum—to the alimentary loop, and the ileum—to the common loop.

Microscopic tissue analysis in the HFHS pigs showed histopathological changes that are characteristic for obesity/diabetes, including fatty liver, reduced size of the pancreatic islets, and low-intensity inflammation in the intestinal mucosa in comparison to the control group (Fig. 2). These changes were partially reversed following the bariatric surgery, as the effects of the long-term HFHS diet intake, such as the adipocyte infiltration in the liver and pancreas (Fig. 2A, Table 1) were still visible. Microscopically, the high-energy diet (HFHS) feeding was associated with reduction of the height of the intestinal villi in all of the examined segments of the small intestine, in comparison the animals fed normal diet, whereas the thickness of the tunica muscularis was modified in the duodenum and ileum only (Table 2). Also, the number of intraepithelial leukocytes was significantly higher in HFHS pigs. The number of goblet cells in the villi was significantly reduced in the ileum concomitantly with a reduction in the size of the Peyer’s patches (Table 2, Fig. 2B). The changes in the IEL count, the number of the goblet cells, and the size of the Peyer’s patches may deteriorate the mechanical/immunological barrier of the gut. The pancreas of the HFHS pigs was characterized by a reduced size of the Langerhans islets in comparison to the control pigs (Table 2).

**Table 2:** Histometric analysis of the small intestine, pancreas, and adipose tissue in pigs fed control diet (C, n = 6), high energy diet (HFHS, n = 6), and high energy diet followed by bariatric surgery (BAR, n = 6).

	C	HFHS	BAR	*p*-value
*Duodenum*				
Thickness of mucosa (µm)	1044 ± 29^a^	1102 ± 64^a^	623 ± 61.6^b^	< 0.0001
Height of villi (µm)	854 ± 42^a^	723 ± 62^b^	403 ± 23^c^	< 0.0001
Thickness of muscle layer (µm)	746 ± 41^a^	577 ± 58^b^	735 ± 172^a^	0.0276
Number of IELs/100 cells (%)	16.3 ± 3.4^a^	27.4 ± 3.3^b^	15.7 ± 2.7^a^	0.0001
Number of goblet cells /100 cells (%)	10.5 ± 2.9	6.9 ± 1.8	8.3 ± 2.3	NS
*Distal jejunum*				
Thickness of mucosa (µm)	942 ± 61	922 ± 99	825 ± 73	NS
Height of villi (µm)	781 ± 61^a^	654 ± 36^b^	565 ± 48^c^	0.0001
Thickness of muscle layer (µm)	369 ± 26^a^	429 ± 56^a^	758 ± 96^b^	< 0.0001
Number of IELs/100 cells	28.6 ± 3.8^a^	56.2 ± 4.2^b^	14.6 ± 1.7^c^	< 0.0001
Number of goblet cells/100 cells	6.7 ± 4.3	4.6 ± 2.1	4.9 ± 1.5	NS
*Ileum*				
Thickness of mucosa (µm)*	911 ± 42^a^	683 ± 65^b^	859 ± 80^b^	0.0001
Height of villi (µm)	587 ± 43^a^	497 ± 47^b^	671 ± 66^c^	0.0002
Thickness of muscle layer (µm)	834 ± 93	781 ± 62	923 ± 164	NS
Average size of Peyer’s Patches (mm^2^)	0.68 ± 0.098^a^	0.58 ± 0.11^a^	0.44 ± 0.097^b^	0.0093
Number of IELs/100 cells (%)	33.0 ± 1.7^a^	42.2 ± 7.8^b^	21.8 ± 0.8^c^	0.0001
Number of goblet cells/100 cells (%)	21.0 ± 3.0^a^	9.9 ± 1.3^b^	9.6 ± 2.1^b^	0.0001
*Pancreas*				
Average size of pancreatic islets (µm^2^)	17,753 ± 1643^a^	13,261 ± 1444^b^	14,662 ± 1748^b^	0.0027

In the pigs treated with the bariatric surgery (BAR), the duodenum corresponds to the enzymatic loop, the distal jejunum—to the alimentary loop, and the ileum—to the common loop. IEL—intraepithelial leukocytes. (Mean and SD, One-way ANOVA, Tukey’s post-test).

*With the layer of Payer’s patches.

One month following the bariatric surgery, the pancreato-biliary diversion had led to a marked reduction of the villi size and thickening of the duodenal mucosa. One month after the bariatric surgery, the IEL number in the duodenum returned to the counts observed in the control pigs (Table 2, Fig. 2B). Following the bariatric surgery, the intestinal villi in the feed loop (corresponding anatomically to the distal jejunum in the non-operated animals) were significantly shorter, whereas the muscularis was almost 1.8-fold thicker, in comparison with the HFHS pigs. The IEL numbers were fourfold reduced after the bariatric surgery in comparison with the numbers found in the HFHS pigs. In the common loop (ileum), the thickness of the mucosa increased due to the longer villi, in comparison to the morphology of the pre-bariatric insulin resistant mucosa, whereas the size of the Peyer’s patches and the IEL counts were reduced (Table 2, Fig. 2B). Also, one month after the bariatric surgery the sizes of the pancreatic islets were not different than in the HFHS pigs.

### Differential protein expression analysis

#### Ileum

**Figure 3:**
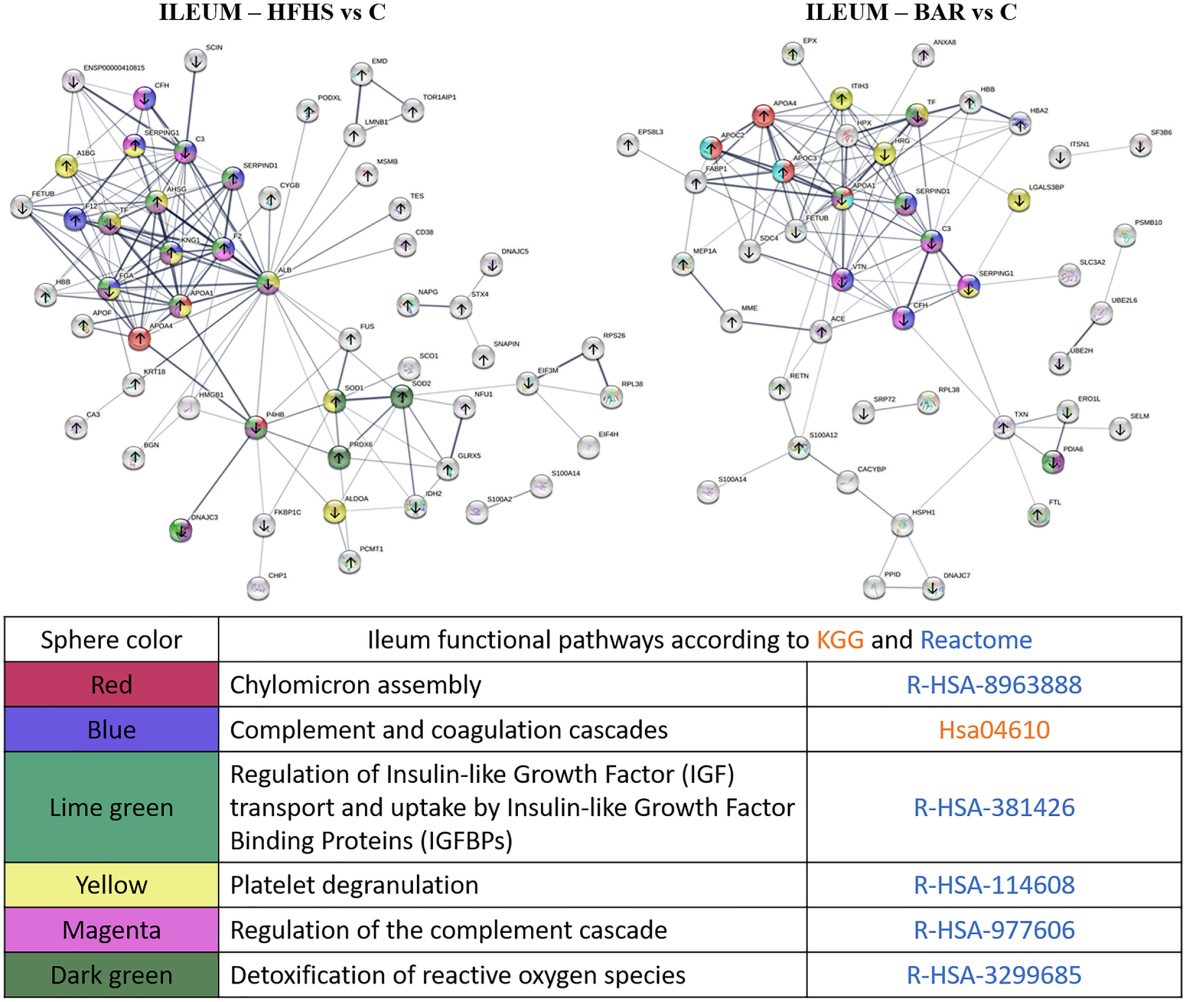
Evaluation of ileum proteome changes unther the influence of high-energy diet (left) and bariatric surgery (right). The ileum functional pathways in the BAR and HFHS pigs according to the KGG and Annotated Keywords (UniProt). Left: high energy diet (HFHS) vs controls (C). Number of nodes: 69; number of edges: 152; average node degree: 4.41; PPI enrichment *p*-value: < 1.0e-16. Right: high energy followed by bariatric surgery (BAR) vs controls (C). Number of nodes: 66; number of edges: 118; average node degree: 3.58; PPI enrichment *p*-value: < 1.0e-16. Arrows up and down show regulation of expression in the HFHS or BAR ileum in comparison with controls (C). *Protein detected only in the HFHS or BAR ileum.

Analysis of the proteome of ileal mucosa showed a moderate number of changes between the proteomic profile of the HFHS pigs and the controls—24 proteins showed negative and 59 positive fold change, versus controls (Fig. 3, Supplement Fig. 1A). Rather than returning to a control profile, the bariatric surgery further modified the protein profile in the ileal tissue. Thus, the bariatric profile of the ileum differed significantly in altogether 80 proteins. In the HFHS ileum, changes (in relation to the control group) were detected in the group of proteins related to the oxidative stress response, such as peroxiredoxin-6 (fold change =  + 1.18), superoxide dismutase (Cu–Zn and Mn) (fold change =  + 1.17 and + 1.19), and carbonic anhydrase (+ 2.23). In the HFHS ileum, modifications in the groups of proteins important for reducing the chylomicron assembly were observed. Especially marked were increased levels of apolipoprotein A-1 (fold change =  + 1.71) and apolipoprotein IV (fold change =  + 3.03) and decreased level of disulphide-isomerase (fold change = − 1.54). The analysis also showed elevated level of blood coagulation factors, such as prothrombin (fold change =  + 1.38), coagulation factor XI (fold change =  + 1.86), and kininogen 1 (fold change =  + 1.52). The highest change, more than fourfold increases, was observed in the levels of two proteins: podocalyxin and the 40S ribosomal protein S26.

In the BAR ileum, the expression of the fat binding proteins increased 16-fold in comparison to the controls reflecting adaptation to the increased amount of fats reaching the surgically modified ileum of the bariatric pigs. These changes are associated with a ca threefold increase in the expression of dipeptidase as well as a 4–5.5-fold increase in the expressions of apolipoproteins C-II and C-III and apolipoprotein I. In contrast to its increased levels found in the HFHS animals, 1.6-fold reductions in the level of apolipoprotein A-I was observed after the bariatric surgery. Moreover, the dcrease in the expression of the heat shock proteins HSP40 and HSP110 (2.4- and 1.7-fold, respectively) indicate that the tissues may be affected by less stressful stimuli following the bariatric surgery.

#### Pancreas

**Figure 4:**
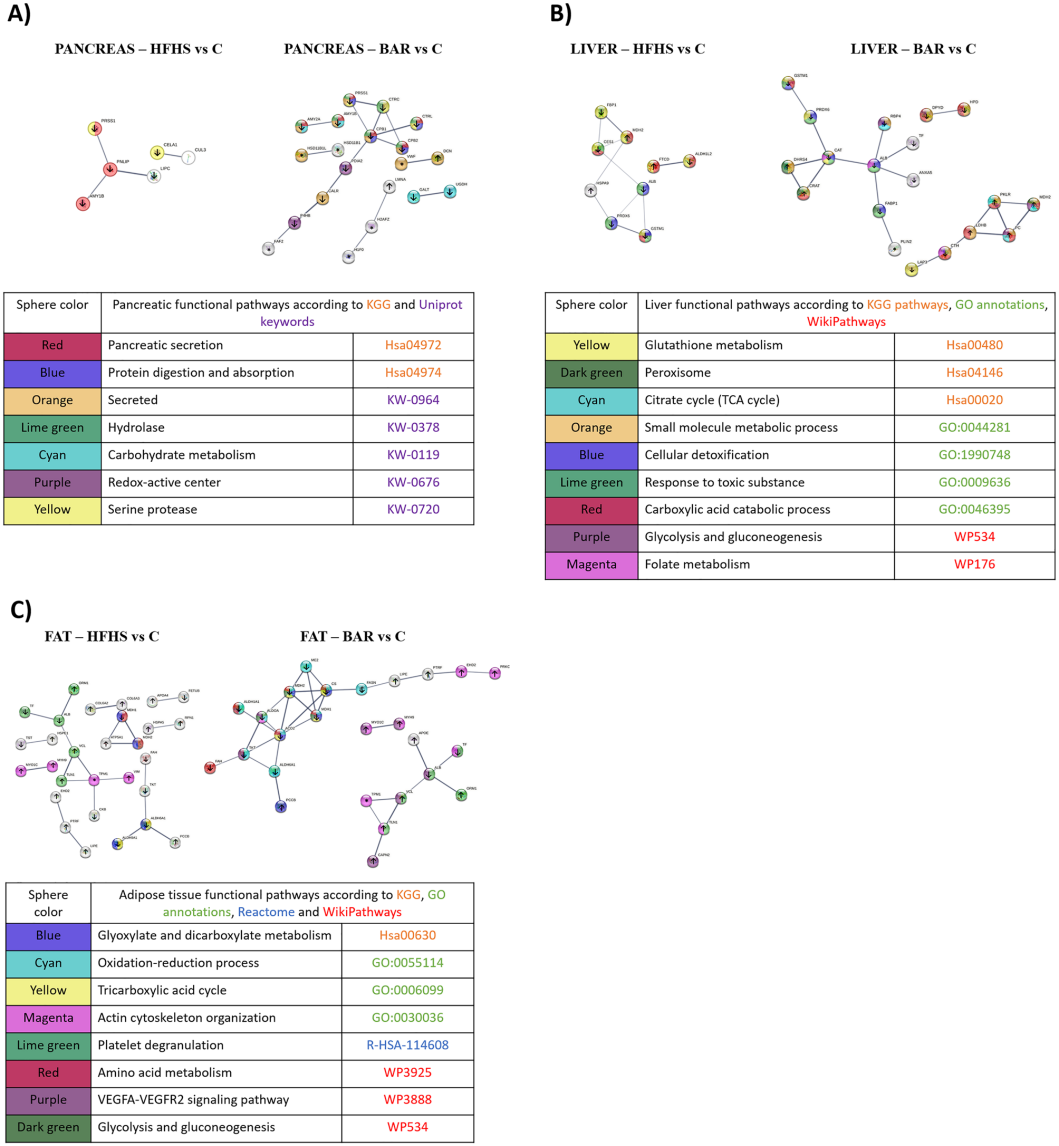
Tissue specific proteome changes under conditions of high-energy diet or after bariatric surgery. Top: The tissue specific functional pathways in the BAR and HFHS pigs according to the biological process (gene ontology), KGG, reactome pathways, Wiki Pathways, and Annotated Keywords (UniProt). Bottom: Descrption of specific pathways coded by colors. (**A**) Pancreas. Top left. High energy diet (HFHS) *vs* control. Number of nodes: 25; number of edges: 3; average node degree: 0.24; PPI enrichment *p*-value: <  < 0.0525. Top right. High energy diet followed by bariatric surgery (BAR) versus control. Number of nodes: 48; number of edges:17; average node degree: 0.708; PPI enrichment *p*-value:1.1^e-08^. Arrows up and down show regulation of expression in the HFHS or BAR group in comparison with the C group. *Protein detected only in the HFHS or BAR group. (**B**) Liver. Top left. High energy diet (HFHS) *vs* control (C). Number of nodes: 21; number of edges: 10; average node degree: 0.762; PPI enrichment *p*-value: < 1.62^e-05^. Top right. High energy followed by bariatric surgery (BAR) *vs* control (C). Number of nodes: 27; number of edges: 19; average node degree: 1.41; PPI enrichment *p*-value: < 1.83^e-07^. Arrows up and down show regulation of expression in the HFHS or BAR group in comparison with the C group. *Protein detected only in the HFHS or BAR group. (**C**) Adipose tissue. Top left: High energy diet (HFHS) *vs* control (C). Number of nodes: 50; number of edges: 22; average node degree: 0.88; PPI enrichment *p*-value: < 1.37^e-07^. Top right: High energy followed by bariatric surgery (BAR) vs control (C). Number of nodes: 52; number of edges: 32; average node degree: 1.23; PPI enrichment *p*-value: 4.56^e-13^. Arrows show up and down regulation of expression in the HFHS and BAR group in comparison with the control (C) group. *Proteins detected in HFHS or BAR group only.

In the pancreases of the HFHS group we found only 31 proteins with changed expression in relation to the controls (16 of them were specific for HFHS only). In the BAR group, 33 proteins were changed, increased or decreased, in comparison to the controls. In this group, 10 proteins showed increased expression, 23, decreased expression, and 19 of the changes were specific only for the BAR group (Fig. 4A, Supplement Fig. 1B). In the HFHS animals all the changed proteins were identified as enzymes with hydrolase activity, such as carboxylic ester hydrolase. Three of them: pancreatic alpha-amylase (AMY2A), pancreatic triacylglycerol lipase (PNLIP), and trypsin-1 (PRSS1) are pancreatic secretory proteins with reduced expression. A markedly decreased expression of pancreatic enzymes, such as trypsin-1 (PRSS1), amylase (AMY1 and AMY2), chymotrypsin C (CTRC), and carboxypeptidase (CPB1 and CPB2) was still observed after the bariatric surgery (Fig. 4A). This is a logical consequence of the removal of stimulation from the duodenum and the activation of the ileal brake by the vagal nerve.

#### Liver

In the livers of the HFHS group only 10 proteins were changed in comparison to the controls (Fig. 4B, Supplement Fig. 1C). The following proteins showed decreased expression: liver carboxylesterase (CES1, fold change = − 1.39), fructose-1,6-bisphosphatase 1 (FBP1; fold change = − 2.31); serum albumin (ALB; fold change = − 2.01); peroxiredoxin-6 (PRDX6; fold change = − 2.1), glutathione S-transferase Mu 1(GSTM1; fold change = − 3.38), mitochondrial 10-formyltetrahydrofolate dehydrogenase (ALDH1L2); and dimethylaniline monooxygenase [*N*-oxide-forming]. The following proteins showed elevated expression: formimidoyltransferase-cyclodeaminase (FTCD), mitochondrial malate dehydrogenase (MDH2; fold change = − 6.66) and mitochondrial Stress-70 protein (HSPA9; fold change = − 1,77). All these proteins are involved in detoxification, toxic stress response, and metabolic processes involving carboxylic acids. The bariatric surgery did not modify the direction of these changes. Interestingly, bariatric surgery increased the expression of proteins involved in glycolysis and gluconeogenesis, folate metabolism, and the citrate cycle (TCA), which may cause carbohydrate metabolism activation in the liver (Fig. 4B).

#### Adipose tissue

In the adipose tissue of the HFHS pigs, we observed impaired action of the protein pathways involved in platelet degranulation, ion transport, and actin filament-based movement, such as cell adhesion and fusion (Fig. 4C, Supplement Fig. 1D). The proteins involved in these pathways: serotransferrin (TF), alpha-1-acid glycoprotein 1 (ORM1), serum albumin (ALB), vinculin (VCL), and talin 1 (TLN1), did not changed even after the bariatric surgery. The highest increase in the level of protein expression in both groups was found in the case of propionyl-CoA carboxylase (PCC), over 14-fold in the HFHS group, and fivefold in the BAR group in comparison to the controls. Decreased expression of proteins observed after the bariatric surgery involved the enzymes of gluconeogenesis, glycolysis, and tricarboxylic acid cycle as well as malate dehydrogenase 2 (MDH1 and MDH2), citrate synthase (CS), NAD-dependent malic enzyme (ME), and aconitate hydratase (ACO2). On the other hand, a clear-cut activation of the pathways involved in the hydrolysis of stored triglycerides to free fatty acids and the internalization and expression of the GLUT 4 receptor (hormone-sensitive lipase—LIPE), caveolae-associated protein 1 (PTRF), EH domain-containing protein 2 (EHD2), and caveolae-associated protein 3 (PRKC) were found in the BAR group (Fig. 4C).

### Influence of diet and/or bariatric surgery on the level of DNA base modifications

**Figure 5:**
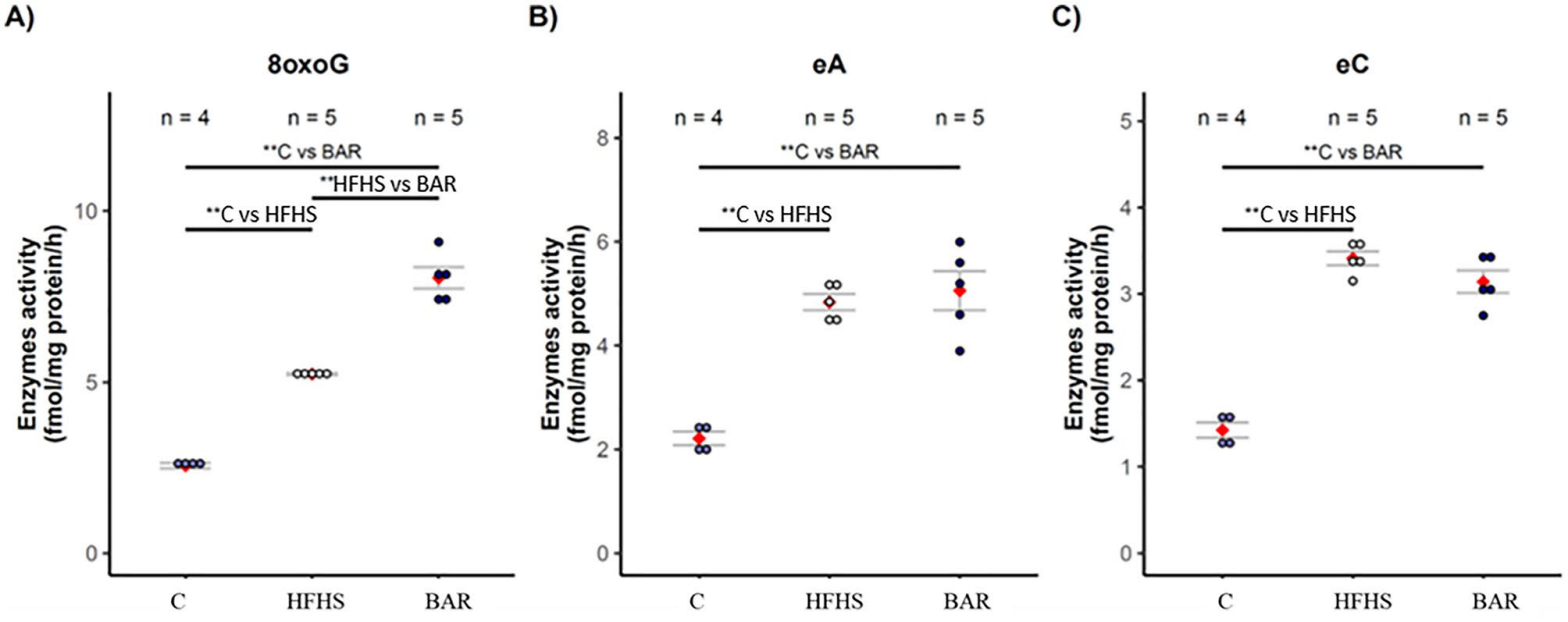
The concentration of oxidative DNA damage and repair enzymes (fmol/mg protein/h) in the mid-jejunum of pigs fed the control diet (C), high energy diet (HFHS), and after bariatric surgery (BAR). Data are represented as points and means (red), with the whiskers (grey) showing sample standard deviation. n—number of samples. **p < 0.01, two‑tailed unpaired Student’s *t* test with Benjamini–Hochberg adjustment (software R version 3.3.0).

Significant changes were observed in the metabolic pathways associated with the modifications of the nucleobases in the tissues. Pig mid-jejunum’s exposure to the HFHS or BAR conditions resulted in elevated level of DNA base modifications: guanine into 8-oxoguanine (8oxoG), adenine into etheno (ε) adenine (εA), cytosine into etheno (ε) cytosine (εC), inducing enzymes involved in base excision repair (BER). Estimation of the expression activity of DNA repair enzymes based on the efficacy of the excision of modified bases showed that the pig exposure to stressful conditions (HFHS, BAR) increased activities of the enzymes repairing εA, εC, and 8-oxoG in comparison to the controls (Fig. 5) as measured by the nicking of an oligodeoxynucleotide with a single base modification^20^. The abundance of the excised εA, εC, and 8-oxoG residues decreased (*p* < 0.001) in the BAR group in comparison to the HE group, but was still higher (*p* < 0.001) than in the control group.

**Figure 6:**
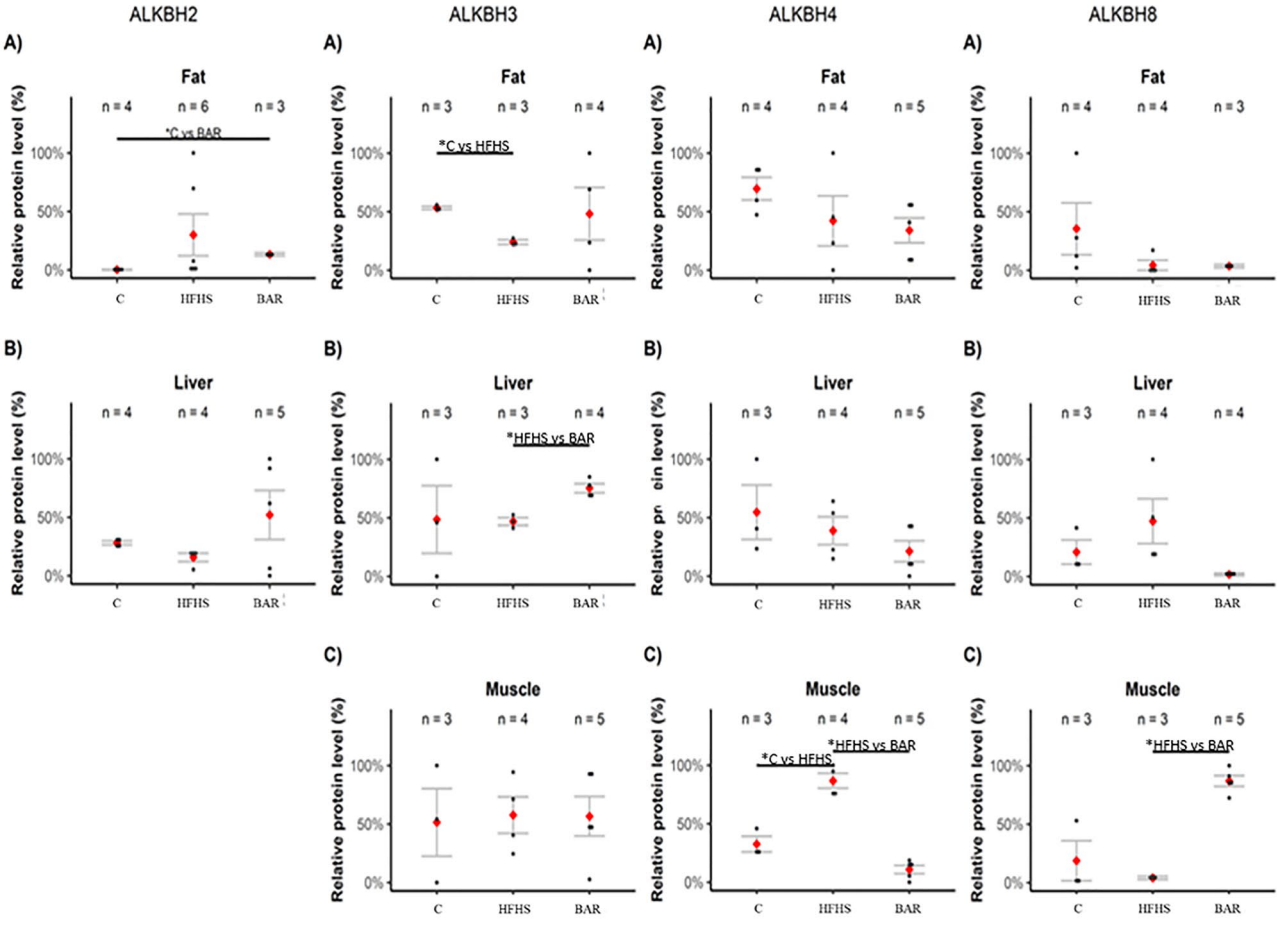
Post-western blot densitometry analysis of the ALKBH 2, 3, 4 and 8 protein level in selected pig tissue. C—Standard diet (n = 6); HFHS—high energy diet with developed obesity and type 2 diabetes (n = 4); BAR—Pigs after bariatric surgery (n = 6). Data are represented as points and means (red), with the whiskers (grey) showing sample standard deviation. n—number of samples. *p < 0.05, two‑tailed unpaired Student’s *t* test with Benjamini–Hochberg adjustment (software R version 3.3.0).

In addition to the BER pathway involved in DNA base modifications repair, some members of the ALKBH family proteins take also part in direct removal of alkylation lesions from DNA and RNA. Post-western blot densitometry analysis was performed for six members of the ALKBH family, namely the ALKBH1, 2, 3, 4, 5, and 8. Increased levels of these specific ALKBH proteins were noted in adipose tissue, liver, and muscle (Fig. 6). Changes in the ALKBH levels were dependent on tissue type, diet, and the presence / absence of bariatric surgery. In the adipose tissue, the ALKBH2 level increased after the bariatric surgery in comparison to the control group (*p* < 0.05). In contrary, the ALKBH3 contents decreased in the pigs fed the HFHS diet (*p* < 0.05). Moreover, its level was higher in the liver of pigs after bariatric surgery than in the HFHS pigs (*p* < 0.05). Changes in the levels of two proteins, ALKBH4 and ALKBH8 were observed in muscle tissue. The level of the ALKBH4 protein increased in pigs fed with high energy diet (*p* < 0.05) and decreased after the bariatric surgery (*p* < 0.05). The level of the ALKBH8 protein was elevated after the bariatric surgery (p < 0.05). No changes in the levels of the ALKBH 1 and 5 were observed in any of the analysed tissues nor in the samples from the pancreas, where no changes were found in any of the ALKBH proteins tested (Supplement Fig. 2).

## Discussion

Here, for the first time, we showed that feeding young pigs for several months a high fat/high sugar diet (150% of energy requirements) may lead to the development of type 2 diabetes and the coincidental modifications in the morphology and proteomic profiles of the intestine, liver, pancreas, and adipose tissue. One month after the bariatric surgery we observed reversal of glucose concentrations in the blood, and to a certain degree in tissue morphology, but not in the proteomic profiles of the investigated tissues nor in the expression of the AKLBH proteins.

### Inducing type 2 diabetes in young obese pigs by high fat /high sugar diet as a model for future research

Our model of type 2 diabetes was developed by feeding young pigs an extreme diet that provided 150% of their nutritional energy. The only manipulation was selecting young pigs with high plasma cholesterol levels (> 95 mg/dl) into the C and HFHS groups. The high energy diet led to the development of type 2 diabetes as confirmed by the increase of triglycerides and glucose in the blood plasma concomitant with characteristic chronic systemic signs (elevated WBC)^21–23^ and local tissue inflammation (rise in oxidative stress and DNA damage)^4^. Several months were required to develop T2D in the experimental pigs, mimicking the situation of overweight and obese children gaining insulin resistance at young age^24^. Pig models have been used in studies of insulin resistance and diabetes^14^, however, most of them relay on the genetic predisposition to develop T2D by the Gottingen and Yucatan pigs or on damaging the beta cells with streptozotocin or alloxan^14,25,26^. This may be an important limitation in studies searching the pathomechanism of insulin resistance and its treatment. In insulin resistance syndrom, the secretion of the insulin from the pancreas may be normal or even elevated due to the decreased negative feedback if the glucose level is not regulated to optimal concentrations. In humans, depletion of pancreatic secretion takes time and is rather a consequence of long-term unregulated level of glucose when it is untreated^27^. In this case even bariatric surgery as a method of obesity and TD2 treatment is less effective^28^.

The advantage of our approach is the use of the commonly available Landrace crossbreeds with confirmed hypercholesterolemia. We believe that our approach, based on an excessive energy intake, resembles better the development of obesity and insulin resistance in humans than the other pig models. Feeding very young pigs for several months a diet exceeding to 150% their energy requirements resulted in structure modification of the gut, liver, and pancreas characterized by a marked adipocyte infiltration, long-lasting low-grade inflammation in the tissues (e.g., increased IEL count in the small intestine), decreased pancreatic inlets size, as well as increased level of the markers of oxidative stress and DNA repair in the tissues. These changes, together with elevated blood glucose and triglyceride levels, as well as the results of the oral glucose load test (OGTT) indicated the development of type 2 diabetes. The only difference between our pig model and the human type 2 diabetes was the lack of cholesterol changes in the blood, although the pigs used in the study showed spontaneous hypercholesterolemia^29^ already at the onset of the high energy diet. Our type 2 diabetes model was developed using a diet rich in rapeseed oil and saccharose only; it required, however, several months to develop stable diabetes mellitus, and was successful in 71%. Other models, based on genetic predisposition of Gottingen or Yucatan pigs to develop T2D or on damaging the pancreatic β-cells with streptozotocin or alloxan (T1D models), may introduce several artifacts into the animal model or manifest only some symptoms of insulin resistance^14,25,26^, leading thereby to improper conclusions. The best evidence that our model could be used to mimic the pathophysiology of T2D in humans is that the diet induced insulin resistance disappeared after the bariatric surgery exactly like in morbidly obese insulin resistant human patients^30,31^. The limitation of our model is the long time to achieve the goal of T2D. The first sings of a pre-diabetic stage were observed after three months, but it was unsteady, until a “solid” diabetes mellitus was achieved a few weeks later. At this time also the weight of the animals may be problematic, as handling animals weighing 150–200 kg needs special equipment and, for example, the drug testing may be considered expensive.

### The consequences of high fat/high sugar diet in pig tissues

The structural tissue changes due to an energy overload the characteristic features for T2D were observed at the structural and molecular level. In the small intestine, the high energy intake shortened the length of the villi, reduced the thickness of the intestinal mucosa, and increased fat deposition in the liver and pancreas tissues. The size of the pancreatic islets was reduced by 25%, showing the impact of glucotoxicity^32^ with a significant decrease in the production of enzymes, such as chymotrypsin and amylase. Simultaneously, the signs of oxidative stress and chronic inflammation, e.g. the elevated IEL in the intestinal epithelium, the increased level of WBC in the blood, the increased concentrations of oxidative DNA damage markers (εA, εC, and 8-oxoG), and DNA repair enzymes in the tissues were observed.

The reduction in islet size in HFHS pigs contrasted with earlier human studies^33,34^. We have no clear explanation for the discrepacies. Nevertheless, we speculate that, our pigs received high fat diet since the very early stage of their life, just two weeks after the switch form mother’s milk to solid feed. The HFHS diet resulted in altered body weight gains as well as on the growth and development of the internal organs involving pancreas. Kehm et al.^35^ and Jo et al.^36^ showed that the size of pancreas islets (as well as the number of β-cells in the islet) increased with age in mice ^35,36^. We think that the long lasting oxidative stress associated with HFHS diet that led to obesity and diabetes mellitus, altered the islet growth and finally resulted in ca 25% reduction of islet’s size as compared to control pigs. Oxidative stress is known to decrease proliferative and regenerative capacity of the tissues.

It has been reported that the pig’s intestine is very susceptible to oxidative stress and may easily succumb to cell or DNA damaging processes^37,38^. Our results showed the DNA repair events associated with the induced within the base excision repair (BER) system MPG, TDG, and OGG1 enzymes take place in the intestine. The analysis and processing of stressful stimuli in the intestine leads to the modification of the activity of other regions of the digestive tract. The highest repair activity observed in all the examined groups was for the 8-oxoG. This may be due to the OGG1 turnover on the damaged DNA, increasing the excision of the 8-oxoG lesions^39^. The use of Fpg protein known as 8-oxoguanine DNA glycosylase removes a broad spectrum of oxidized and alkylated bases from double stranded DNA e.g. 8-oxoguanine, 8-oxoadenine, unsubstituted and substituted imidazole ring-opened purines introduced into DNA by hydroxyl radicals (e.g., FapyG, FapyA). The Fpg protein shows two additional activities: (i) The AP-lyase activity removing apurinic/apirimidinic (AP) site and leaving a 1 base gap by β-δ-elimination and (ii) a dRPase activity removing the 5′ terminal deoxyribose phosphate from DNA incised by an AP endonuclease. DNA damage above 3% by Fpg protein is a strong genomic DNA damage to all oxidized and modified guanines occurring as 8-oxoguanine, fapy-adenine (FapyA), and fapy-guanine (FapyG)^37–46^.

The 8-oxoG has been established as an important biomarker of oxidative stress^40,46^ cancer risk^41–43^ the ageing processes, including degenerative diseases^44,46^ and as a general biological marker of lifestyle and diet^45,46^. Several studies reported an elevated risk of the development of some type of cancers in insulin resistant patients due to the increased levels of oxidative stress, increased DNA damage, or chronic inflammation, which we have also observed in our model. Here, we found more than fourfold elevated level of podocalixin in the intestines and more than sixfold decreased level of 10-formyltetrahydrofolate dehydrogenase in the HFHS pigs. This may constitute additional prognostic markers of cancer development predisposition^47,48^.

The role of the small intestine in the development of insulin resistance and diabetes mellitus was described previously. The gut participates in the dysregulation of glucose metabolism on many levels involving induction of low-grade inflammation in the gut mucosa, modification of the microbial milieu and the intestinal hormones secretion (e.g., peptide YY and glucagon-like peptide-1, GLP-1), activation of the lipopolysaccharide TLR-4 axis, and modulation of the integrity of intestinal barrier^49^. The proteomic analysis of the HFHS ileum showed increased activation of the platelet degranulation pathways, which may result in developing macro- and micro-angiopathies leading to progressive deterioration of the connections between enterocytes, increased permeability of the epithelial barrier, and inflammation in the intestine^50^. Other studies pointed out a link between dysbiosis of the gut microbiota and enhanced permeability and local inflammation of the intestines in TD2 patients^51,52^. Recently, Wang and co-workers^53^ confirmed their observations on T2D Chinese hamsters model, where the activation of platelet degranulation caused local inflammation in the intestine. The platelet degranulation pathway was also significantly changed in the adipose tissue. This, along with endothelial damage, enhanced coagulation, and impaired fibrinolysis was previously found in obese humans as an important part of the mechanism currently recognized to increase arteriosclerosis and arterial thrombotic risk in obese and diabetic subjects^54,55^. Additionally, in the liver, adipose tissue, and ileum of the HSHF pigs, we also found a significantly decreased level of albumin. In previous studies, low level of albumin was associated with the metabolic profile containing increased adiposity, increased plasma glucose concentrations, and adipose tissue inflammation. The level of albumin, was shown to correlate strongly with the state of insulin resistance. It also influences diabetes mellitus as coronary heart disease and arteriosclerosis protein^56–58^.

Previously, we have found that FTO protein performs several distinct functions depending on its localization^5^. Especially intriguing is the pancreas where FTO is not localized in the whole organ but exclusively in the β-cells. This could indicate the importance of the process of demethylation, performed by the FTO demethylase, played in the insulin metabolism. In the present study, we determined the levels of other proteins of the ALKBH family, namely the ALKBH 1–5 and 8, and found the levels of the ALKBH2, ALKBH 3, ALKBH 4 and ALKBH 8 changes in the pigs on the HFHS diet in adipose tissue, liver, and muscle in comparison to the pigs on the control diet. This indicates that the HFHS diet leads to an increased number of the DNA/RNA alkylation lesions inducing levels of the mentioned dioxygenases. The ALKBH2 protein was localised to the nuclei^59^ exhibiting similar enzymatic activity as the *Escherichia coli* AlkB (EcAlkB) protein removing alkylation lesions from DNA and RNA. It prefers acting on double-stranded DNA (dsDNA) and the main substrates are methylated adenine (1-meA) and cytosine (3-meC)^60–62^^.^ It was also discovered that the ALKBH2 protein is able to remove larger lesions, like etheno adducts, from DNA and is involved in the oxidation of 5-methylcytosine (5-meC)^62–65^. Like ALKBH2, ALKBH3 removes the methyl group from the 1-meA and 3-meC in DNA. However, ALKBH3 prefers lesions occurring in single-stranded DNA (ssDNA) over dsDNA as a target. It also repairs damage found in RNA^59–61^. Subsequent studies have shown that deficiency of the ALKBH8 increases the level of reactive oxygen species, enhancing thereby cellular aging and negatively affecting mitochondrial functions^66^. Preservation of the mitochondrial functions may be the aim of the elevated level of the ALKBH8 in pigs after the bariatric surgery in comparison to the pigs on the HFHS diet, demanded by the drastic changes in the cellular power management induced by the surgery and the changed diet. The changes in the levels of proteins capable of scavenging the etheno adducts suggest that the stress conditions induced by the HFHS diet affect the entire body. They include cellular and nuclear compartments as well as nucleic acids, which may cause dysregulation of the existing metabolism at the level of RNA, but also lead to permanent DNA damage leading to the induction of oxidative stress in the cell.

### Consequences of bariatric surgery observed in the tissues

Autopsy performed one month after the bariatric surgery confirmed the tightness of the connections of the intestinal loops and the absence of adhesions or local inflammation in the modified gastrointestinal tract and the peritoneum. One month after the surgery we noticed normalized glucose level and a decreased level of triglycerides along with a lowered level of WBC in the blood. This explains the increase in the insulin sensitivity of the tissues. Surprisingly, we observed very few changes on structural and molecular level in the organs crucial for the glucose metabolism. The main change on the structural level included a significant decrease in the IELs counts, an increase in the length of the ileal villi, and reduced size of the Peyer’s patches. In general, the decreased level of inflammatory signs may be an answer to the GIT remodelling after the bariatric surgery and the modification of the microbiota composition playing a significant role in the local inflammation of the intestine affecting the insulin resistance in obese and diabetic patients^67^. On the molecular level, the most important changes improving the insulin sensitivity in the operated pigs was a significant reduction of the oxidative stress^68^.

The analysis of the ileal proteome revealed elevated expression of proteins involved in the methylation processes after a high energy intake in comparison to the control, which may lead to epigenetic modifications characteristic of T2D^69^. In our study, the number of proteins involved in methylation insensibly decreased by the bariatric surgery in pigs, but human data showed that after a Roux-en Y gastric bypass, DNA methylation was still observed in the human blood even six months after the surgery and was linked to the involved genes^70^. Interestingly, we observed that the Roux-en Y gastric bypass promotes epigenetic changes in specific pathways, mainly the ones related to inflammation, angiogenesis, and endothelin-signalling, which we also found to be crucial to the ileal phenotype of insulin resistance. In the HFHS group, we found reduced level of secretory proteins: pancreatic alpha-amylase (AMY2A), pancreatic triacylglycerol lipase (PNLIP), and trypsin-1 (PRSS1) in the pancreas. After the bariatric surgery, we still observed a decreased expression of the pancreatic enzymes with no other signs of improvement in the pancreatic functions on either the structural or molecular level. We postulate that the post-bariatric modification of the exocrine pancreatic function is not crucial for controlling insulin sensitivity and that the regeneration of the pancreas presumably takes more time^71^.

In our investigations of the changes happening among the ALKBH proteins the most evident were the changes in the level of the ALKBH4 protein in the muscles, where it was elevated in the HFHS pigs, but fell to the control levels after the bariatric surgery. The ALKBH4 protein was shown to be involved in actin-myosin structure organization, cleavage furrow ingression, and protein demethylation^72^ indicating that the protein may be involved in the pathomechanism of insulin resistance in the muscles.

### Limitations of the methods

Important limitation is that during our experiment the HOMA-IR test was not performed, and diabetes type 2 was confirmed by clinical sings including obesity, polyuria and polydipsia, as well as hyperglycaemia, hyperlidemia and glucose tolerance (OGGT) test. Also, the idea of using mass spectrometry was to compare whole proteomes of the tissues to check which pathways rather than the single proteins might be affected by the HFHS diet and after the bariatric surgery in reversion of hyperglycemia on the molecular level. Mass spectrometry analysis, however, has limitations in protein detection, especially if it is used for whole proteome screening in the tissue. Presence of regulatory peptides in picomolar concentrations, such as GLP-2, or their precursors in the gut neural and/or endocrine cells might be covered by the other proteins with higher abundance. Moreover, small molecular weight regulatory peptides may by below the detection margin in our method. To detect small size peptides with very low concentrations, e.g. GLP-2, other more sensitive methods of analysis should be performed such as Parallel Reaction Monitoring mass spectrometry (PRM-MS) with particular weight standards or radioimmunoassay (RIA), like it was done in a previous rat study with e.g. plasma and tissue insulin, apelin, PYY and visfatin^10^.

### Conclusions

We developed a pig type 2 diabetes model, where the symptomes are achieved exclusively through diet modification. This model is more reliable than the previously described, dependent on genetic defects or the destruction of pancreatic β-cell with streptozotocin or alloxan, since the mechanism of the insulin resistance may, as in humans, be easily reversed by bariatric surgery treatment. This model may be used for future studies of the mechanisms of diabetes mellitus but also for testing new pharmacological TD2 treatments. Our data indicate histopathological and proteomic changes critical for glucose metabolism and characteristic for the T2D organ phenotype, such as chronic inflammation, increase in oxidative stress and DNA damage, as well as the presence of atherosclerotic and procancerogenic markers. The structural and proteomic profile shows that the rapid improvement of blood parameters after a bariatric surgery does not correspond to the rate of change in the tissues. Although the main processes occurring within one month after the surgery were the reduction of the damage to DNA, oxidative stress, and chronic inflammation, the phenotypes of the bariatric pigs were still associated closer with the HFHS pigs than with the control group. Finally, we have found that the ALKBH family proteins involved in DNA/RNA repair/modification may play important role in the mechanism of the development/protection against insulin resistance, however, more studies on this subject are necessary.

## Materials and methods

### Animals and experimental design

The protocol was conducted in compliance with the European Union’s regulations concerning the protection of experimental animals. The study protocol was approved by the II Local Ethical Committee, Warsaw University of Life Sciences, Warsaw, Poland. A total of 20 male and female pigs (*Sus scrofa domesticus,* crossbreed Polish Landrace 50% ♀ and Duroc 25%, Pietrain 25% ♂) from the university research pig farm were used. The herd was virus-negative for porcine reproductive and respiratory syndrome, mycoplasmosis-negative, and rhinitis atrophicans infectiosa suum-negative, as confirmed by serum ELISA tests, and leptospirosis-negative as confirmed by the microscopic microagglutination test. All piglets were delivered by multiparous sows at term and were clinically healthy without signs of intrauterine growth retardation (IUGR)^73^. Sows were fed a standard diet for pregnant sows (dry matter (DM) 87.6%, metabolizable energy (ME) 11.35 MJ/kg, and crude protein (CP) 12.9%). After farrowing, the diet was switched to the diet for lactating sows (DM 87.3%, ME 12.93 MJ/kg, CP 17.1%). On postnatal days 3 and 17 (PD3 and PD17), all new-born piglets were injected intramuscularly with 100 mg iron dextran (FeDex, Ferran 100, 10% solution, Vet-Agro, Lublin, Poland), and the males were castrated in the first postnatal week. From PD10, the piglets were creep fed ad libitum with prestarter feed (DM 88.6%, ME 13.3 MJ/kg, CP 18.3%). On PD28, the piglets were weaned to a standard commercial starter diet (DM 88.6%, ME 13.2 MJ/kg, CP 18.1%). The feed and water were provided ad libitum.

In this study, a total number of 20 pigs from four litters were used. Six weeks after birth (PD42), 20 pigs with confirmed spontaneous hypercholesterolemia (plasma cholesterol > 95 mg/dl) were divided into two groups. The first control group (C, n = 6) continued to be fed with a starter diet, then with grower (PD56-PD120: DM 88.6%, ME 13.0 MJ/kg, CP 17.0%), grower 2 (PD120-PD180: DM 88.5%, ME 12.7 MJ/kg, CP 16.0%), and finisher (PD180-PD320: DM 88.2%, ME 12.4 MJ/kg, CP 15.0%) diets. The second group was fed with a high fat/high sugar diet (HFHS, 150% of the nutritional energy requirements, n = 14). In the HFHS group, the energy increase was achieved by the addition of sucrose and rapeseed oil to the standard diet. The pigs were adapted to the HFHS diet within 2 weeks. The C and HFHS diets were given for 9 months until slaughter or bariatric surgery. During this time, 10 out of 14 HFHS pigs developed type 2 diabetes as confirmed by pre-prandial blood glucose, triglyceride concentration, and oral glucose tolerance tests (OGTT). All control (C) pigs, and the HFHS pigs that were not subjected to the bariatric surgery were killed by barbiturate overdose (Morbital, 0.3–0.6 ml/kg b. wt., iv, Biowet, Poland) and exsanguinated. Tissue samples were immediately collected from the liver, pancreas, small intestine (duodenum, proximal and distal jejunum, and ileum), and subcutaneous adipose tissue, and frozen in liquid nitrogen or fixed in buffered formalin.

**Figure 7:**
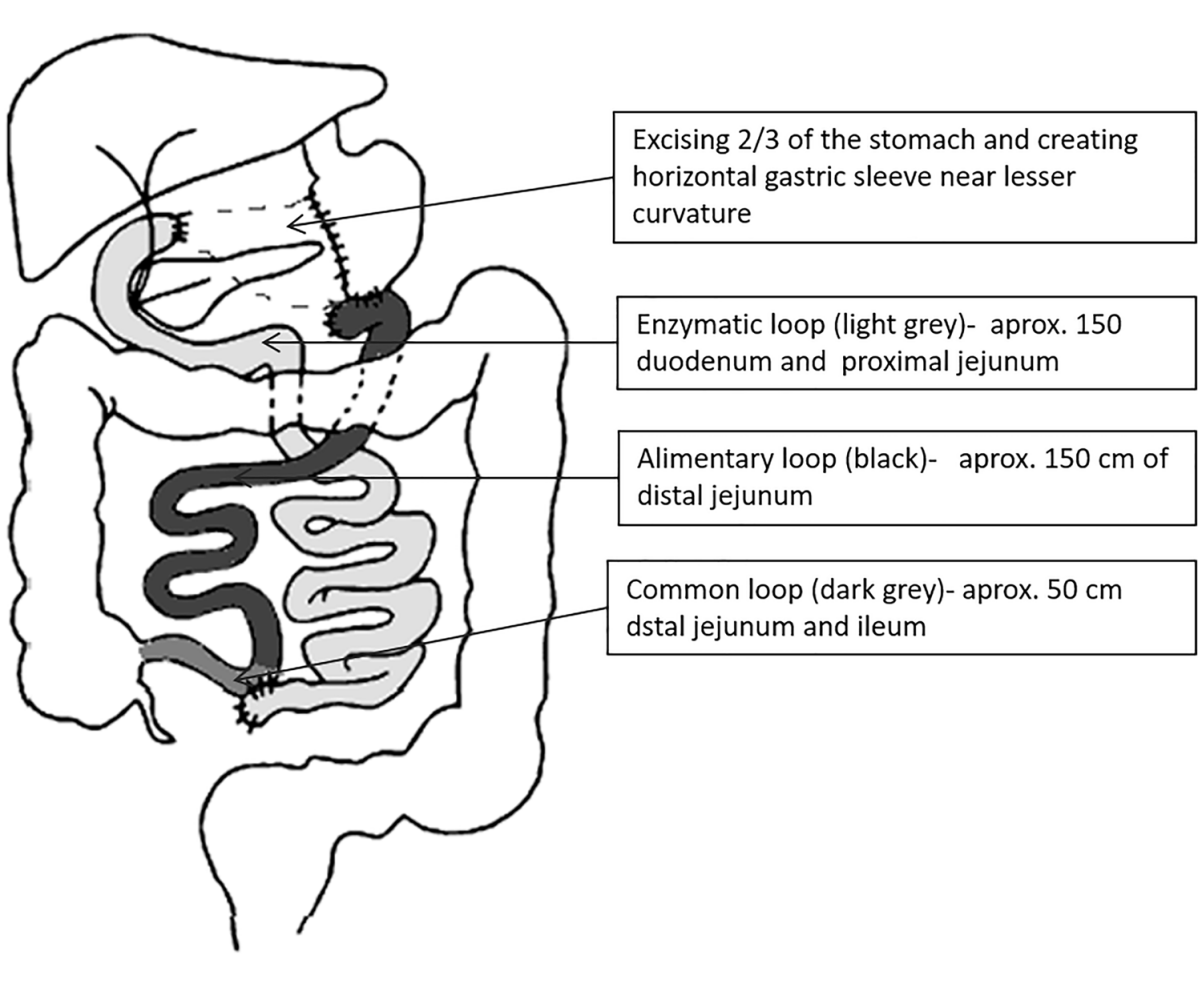
Schematic view of the bariatric surgery in type 2 diabetes pigs according to the modified procedure of biliary-pancreatic diversion (Scopinaro method). The surgery consisted of creating a gastric sleeve and transecting the small intestine 50 cm from the ileo-cecal junction. The proximal end of the duodenum was closed, and the end of the proximal part of the intestine was anastomosed side-to-side to the distal part of the intestine to create a common limb. The distal part of the intestine was anastomosed side-to-side to the stomach to create the alimentary limb. Adapted from Pardela et al.^78^.

Nine months after the introduction of the HFHS diet, 6 of 10 diabetic HFHS pigs underwent bariatric surgery (group BAR, n = 6). In brief, the feed was reduced by half (morning portion) the day before surgery and totally withdrawn on the evening before the surgery and during the next two postoperative days. Starting from the 3^rd^ postoperative day, the animals received water orally and oral feeding was introduced from the fourth postoperative day. The operated pigs received the control feed ad libitum: for the first few days in a soaked form and then in dry form. Azaperone (4 mg/kg b.wt., Stressnil, I.M., Janssen & Cilag Pharma, Vienna, Austria) was given as premedication. General anaesthesia was induced with 5% isoflurane (Aerrane Baxter, Healthcare Baxter Inc., Warsaw, Poland) mixed with oxygen (2 dm^3^/min gas flow) given via an endotracheal tube and was maintained with isoflurane at a concentration of 2% during the entire surgical procedure. The surgery consisted of midline laparotomy followed by a modified Scopinaro procedure^10,74^ consisting of excising 2/3 of the stomach and creating a smaller stomach pouch and transecting the small intestine 50 cm from the ileo-cecal junction. The proximal end of the duodenum was closed and the end of the proximal part of the intestine was anastomosed side-to-side to the distal part of the intestine around 20 cm from the ileo-cecal junction to create a common limb. The distal part of the intestine was anastomosed side-to-side to the stomach to create the alimentary limb (Fig. 7). After the surgery the animals received analgesic, tolfedine (Vetoquinol, 0.1 ml/head, s.c., Biowet, Poland), and antibiotic, Baytril (0.1 ml/head, i.m., Bayer, Germany) treatment for 3 days. One month after the surgery the operated pigs were killed by barbiturate overdose and exsanguinated. The gastric sleeve and the intestinal loops were checked for tightness of anastomoses, and tissue samples were immediately collected from the liver, pancreas, small intestine (duodenum and proximal jejunum—enzymatic loop, distal jejunum—food loop, and ileum—common loop), and subcutaneous adipose tissue, and frozen in liquid nitrogen or fixed with buffered formalin.

### Oral glucose tolerance test

Oral glucose tolerance tests (OGTT) were performed once a month starting from the 4^th^ month of life and continued until slaughter. A bolus of glucose solution (1.75 g/kg b.wt.) was administered by intragastric tube before the morning feeding. Glucose concentration was measured in a drop of blood from the ear vein just before and 1 and 2 h after the oral administration of glucose using a glucometer (One Touch Select® Plus glucometer, LifeScan Europe, Switzerland) and dedicated test strips.

### Blood analysis

Blood for haematology studies was withdrawn from the left external jugular vein in the morning after overnight fasting: one tube with anticoagulants (7.5 ml; EDTA—K2) for morphology studies, and one for biochemical analysis (7.5 ml with silica particles as clot activators). The samples for blood morphology were stored at + 4 °C and analysed within 4 h after collection. The samples for biochemical studies were stored at + 4 °C, centrifuged at 2000 RPM for 10 min within 4 h after collection, and frozen at − 20 °C for further analysis. The blood analysis was performed using the Mindray BC-2800 Vet (Mindray Medical, Poland) apparatus. The measured parameters were: white blood cells (WCB), red blood cells (RBC), thrombocytes (PLT), and haematocrit HCT. Biochemical analyses were performed using the Mindray BS-120 (Mindray Medical, Poland) apparatus. The analysed parameters were: glucose, triglycerides, and total cholesterol.

### Histology analysis

Whole sections of the duodenum, proximal and distal jejunum, and ileum, with a length of at least 5 cm, were immediately flushed with phosphate buffer saline (PBS) at room temperature, placed into 100 ml containers with 4% buffered formalin, and sealed tightly. After 48–72 h the formalin was removed and the samples were incubated with ethanol (70%). The histology samples were stored at room temperature until the next step of histological slide preparation and microscopic analysis. The whole tissue intestinal samples were fixed using a tissue processor (Leica TP1020, Kawa.ska, Zalesie Górne, Poland) (dehydration in increasing concentrations of ethanol, xylene washing, and paraffin embedding). The samples were dissected into 5 μm sections (Leica RM2255 microtome, Kawa.ska, Poland) and processed using the standard Trichrome staining protocol (Multistainer Leica ST5020, Kawa.ska, Poland). The thicknesses of the intestinal mucosa, the muscle layer, the areas of Peyer’s patches in the ileum, and the islets of the pancreas were measured at 4× magnification^70^ using an optical microscope (Olympus BX43, Tokyo, Japan) equipped with a digital camera and the CellSens v.3 (Olympus) software. Only complete sections of the Peyer’s patches were chosen for analysis (both the basal round-shaped part and the top part had to be intact and free of artifacts). The percentage of goblet cells (GCs) in the epithelium and the percentage of intraepithelial leukocytes (IELs) were measured as follows. First, the epithelial lineage area was discriminated on the villus using the area of interest option (MicroImage, Metro Manila, Philippines). Second, the total number of cell nuclei was measured in the marked area of interest (MicroImage). Third, the goblet cells and intraepithelial leukocytes were counted manually in the area of interest. This procedure was repeated on 6 to 10 villi in one slide, and finally, the percentage ratio of goblet cells and intraepithelial leukocytes to all epithelial cells was calculated. For each slide, the choice of the villi for cell counting was based on their structural integrity, profile shape, size, and morphological consistency^75^. The area of the pancreatic islets was measured in 3 slides repetition from one tissue sample by measuring the area of cross-section of the 10 biggest pancreas islets in each slide (Olympus BX43, Tokyo, Japan equipped with a digital camera and the CellSens v.3 Olympus software). The fatty liver was evaluated as the percentage of hepatocytes with fat droplets and it was evaluated qualitatively as fatty liver or not dependent of histopathological picture of hematoxilin eosin stained slides. Total, minumum 10 fields of view on each of 3 slides from each tissue sample were evaluated under the objective 40×.

### Labelling and detection of oligodeoxynucleotides εA, εC and 8-oxoG

The whole tissue samples of the distal small intestine were homogenized with 4 volumes of 50 mM Tris–HCl, pH 7.5 buffer containing 1 mM EDTA and a protease inhibitor cocktail (Sigma). The cells were disrupted by sonication (three 15-s pulses with 30-s intervals). The cell debris was removed by centrifugation (7000 g, 4 °C, 15 min) and the supernatants were collected. The protein concentration was determined by the Bradford method using the protein assay reagent (Sigma). The supernatants were stored at − 80 °C for further analysis. The εAde, εCyt, 8oxoG, and etheno adduct excision activities were measured by the nicking assay using an oligodeoxynucleotide duplex containing a single εAde, εCyt, 8oxoG residue according to Obtułowicz et al.^20^ .

### Proteomic profiling analysis

Sample preparation and mass spectrometry analysis was performed at the Mass Spectrometry Laboratory at the Institute of Biochemistry and Biophysics PAS. Frozen samples of the ileum, pancreas, liver, muscle, and adipose tissues were divided into three groups, C, HFHS, and BAR and procided in several steps: (1.Tissue lysis) Around 1 g of tissue was transferred to 2 ml tubes containing the mix of 1.4 mm and 2.8 mm ceramic beads. The lysis was performed in 400 µl of 50% trifluoroethanol (TFE) in 100 mM triethylammonium bicarbonate buffer (TEAB). The tissue was lysed using Precellys Evolution homogenizer (Bertin Technologies) using 6 rounds of program with the following parameters: 6800 rpm in 10 cycles for 20 s with 20 s pause in between. After homogenisation the samples were sonicated in ice bath for 30 min, centrifuged (14,000 g, 20 min, 4 °C) and transferred to 1.5 ml low-retention tubes. The lysates were diluted to 25% TFE concentration and centrifuged again. The protein concentrations were measured using Pierce BCA Protein Assay Kit (Thermo Fisher Scientific). (2.Proteins digestion) 50 µg of each protein sample was diluted to the final concentration of TFE 6% with 100 mM TEAB buffer and to volume of 50 µl. Cysteine bridges were reduced by 1 h incubation with 5 mM tris(2-carboxyethyl)phosphine (TCEP) at 60 °C followed by 15 min incubation with 20 mM s-methylmethanethiosulfonate (MMTS) at room temperature. Digestion was carried out overnight using trypsin in 1:25 enzyme-to-protein ratio at 37 °C. After digestion samples were diluted to 500 µl with 0.1% formic acid (FA) in water. (3.Mass spectrometry) 1 ug of peptides from each sample was analysed using LC–MS system composed of Evosep One (Evosep Biosystems) coupled to a Orbitrap Exploris 480 mass spectrometer (Thermo Fisher Scientific) via Flex ion source (Thermo Fisher Scientific). Samples were loaded onto disposable Evotips C18 trap columns (Evosep Biosystems) according to manufacturer protocol with minor modifications as described previously^71^. Briefly, Evotips were activated with 25 µl of Evosep solvent B (0.1% FA in ACN) by 1 min centrifugation at 600 g followed by 2 min incubation in 2-propanol. After equilibration with 25 µl of solvent A (0.1% FA in water), 20 µl of each sample solution was loaded onto the solid phase. Bound peptides were washed with 50 µl and covered with 300 µl of solvent A. Chromatography was carried out at a flow rate 250 nl/min using the 88 min (15 samples per day) gradient on EV1106 analytical column (Dr Maisch C18 AQ, 1.9 µm beads, 150 µm ID, 15 cm long, Evosep Biosystems). Data was acquired in positive mode with a data-dependent method using the following parameters. MS1 resolution was set at 60 000 with a normalized AGC target 300%, Auto maximum inject time and a scan range of 300 to 1600 m/z. For MS2, resolution was set at 15 000 with a Standard normalized AGC target, Auto maximum inject time and top 40 precursors within an isolation window of 1.6 m/z considered for MS/MS analysis. Dynamic exclusion was set at 20 s with allowed mass tolerance of ± 10 ppm and the precursor intensity threshold at 5e3 Precursors were fragmented in HCD mode with normalized collision energy of 30%. Spray voltage was set to 2.1 kV, funnel RF level at 40, and heated capillary temperature at 275 °C. (4.Data analysis) Obtained rawdata were pre-processed with Mascot Distiller (version 2.8.1, Matrixscience). Resulting peaklists were searched with Mascot search engine (version 2.8, Matrixscience) against Sus scrofa proteins deposited in Uniprot database (version 2021_04, 49,792 sequences; 29,250,520 residues), with following parameters: enzyme—trypsin, fixed modifications—Methylthio (C), variable modifications—Oxidation (M), peptide mass tolerance—5 ppm, fragment mass tolerance 0.01 Da, instrument type: HCD. Peptide FDR threshold was set to 1%, subset proteins were not included in the final list.

### Bioinformatic analysis of protein–protein interaction

For bioinformatic analysis only proteins with significant difference (*p* < 0.05) between groups were of interest. The software STRING (version 11.5) was used to explore the protein–protein interaction of the protein profile^76^. A stringent, high confidence cut-off of 0.7 was deployed in the minimum required interaction score categories to select only the significant interaction network.

### Western blot of the ALKBH family of proteins

Frozen samples were homogenized in liquid nitrogen and extracted with RIPA buffer (Sigma-Aldrich) supplemented with 50 mM EDTA and 4 mM PMSF in the presence of a protease inhibitor cocktail (Sigma-Aldrich). The cellular debris were spun down and the content of the supernatant’s protein was measured using the Bradford assay (Bio-Rad). Samples were diluted with SDS-PAGE loading buffer to a protein concentration of 2.5–5 μg/μl depending on the tissue type. Then 10 μl portions were loaded onto Mini-PROTEAN TGX 4–15% gradient gels (Bio-Rad). The western blot analysis was performed with specific primary monoclonal and polyclonal antibodies used at dilutions 1:200–500 against ALKBH1, ALKBH3 (Santa Cruz Biotechnology), ALKBH2, ALKBH5, ALKBH6, ALKBH7, ALKBH8 (Sigma-Aldrich) and ALKBH4 (Proteintech) with the appropriate 1:2000 diluted secondary anti-mouse IgG antibody (Sigma-Aldrich) or anti-rabbit IgG antibody (Santa Cruz Biotechnology) conjugated with horse-radish peroxidase. All incubations were performed in 5% milk/PBST (PBST—phosphate buffer saline with 0.1% TWEEN-20). Chemiluminescence was measured using the ChemiDoc MP Imaging System (Bio-Rad) and ImageLab, version 5.0 build 18 software (https://www.bio-rad.com/en-pl/product/image-lab-software?ID=KRE6P5E8Z). The samples were standardized using total protein^73^. Briefly, the total protein content was standardized using four steps: (1) equal amounts of tissue were taken for extraction in RIPA buffer (50 mg); (2) the extracts were assayed for protein content by the Bradford method; (3) equal amounts of the extracts were loaded onto gels and the amounts were verified by Stain-free staining using ImageLab software; (4) the proteins, transferred to nitrocellulose membranes, were visualized by Ponceau-S reversible staining prior to the final Western blot. The primary monoclonal antibodies were verified by performing the silencing of the corresponding ALKBH protein expression in HeLa cell line using small interfering RNA (siRNA) and subsequent analysis by the Western blot method. After using the siRNA, we observed a very weak signal of the invastigated proteins, corresponding to their molecular weight, in comparison with the control.

The intensity of a sample’s chemiluminescence was expressed as the difference between the signal from the area containing the specific protein strand and the background signal from an analogous reference area. Signals from samples located on different blots were compared using normalization against signals from common samples located on separate membranes. Depending on the blot, these were signals from the HEK293 cell line, the “BAR_273_Muscle” common sample (for selected ALKBH3 cases) or the non-specific band of the unstained protein ladder (for selected ALKBH4 cases). The obtained results were normalized against the most intense signal in a given comparison. The generated data were then used in statistical analysis.

### Statistical analysis

Data were analysed using one-way ANOVA followed by the Tukey’s post-test. All statistical analyzes were done using GraphPad Prism v.5.0 (GraphPad Software, San Diego, CA, USA); *p* < 0.05 was considered significant, *p* < 0.01 highly significant, and *p* < 0.1 a trend. For proteomic data, the obtained results were analysed statistically using Statistica 13 software (StatSoft Inc. 2021). Mean values and standard deviations (SD) of emPAI were calculated. Variance analysis (ANOVA) was used to determine *p*-value for each identified peptide in each group of pigs.

### Ethics approval and consent to participate

The protocol was conducted in compliance with the European Union’s regulations concerning the protection of experimental animals. The study protocol was approved by the II Local Ethical Committee, Warsaw University of Life Sciences, Warsaw, Poland No. 7/2015 and No. 8/2015. We confirm that study in manuscript are reported in accordance with ARRIVE guidelines.

### Supplementary Information

Supplementary Information.

## Data Availability

Data generated or analysed during the study are available from the corresponding author by reasonable request.

## Abbreviations

εAde: Etheno deoxyadenosine
εCyt: Etheno deoxycytosine
8oxoG: 8-Oxoguanine
ALKBH: Family of specific demethylases
CP: Crude protein
DM: Dry matter
FTO: Fat mass and obesity-associated protein also known as alpha-ketoglutarate-dependent dioxygenase
HFHS: High fat-high sugar diet
OGTT: Oral glucose tolerance test
T2D: Type 2 diabetes (insulin dependent)
