# Supplementary material for: The proteomic profile is altered but not repaired after bariatric surgery in type 2 diabetes pigs
Source: Sci Rep. 2024 May 3;14:10235. doi: 10.1038/s41598-024-60022-9 (PMC11068747; doi:10.1038/s41598-024-60022-9)
Supplement: Supplementary file 1 — Supplementary Information. [file 41598_2024_60022_MOESM1_ESM.pdf]

Supplement

Is insulin resistance reversed by bariatric surgery? Proteomic studies in pigs fed high energy diet.

Karolina Ferenc[a], Michał Marcinkowski[b], Jarosław Olszewski[a], Paweł Kowalczyk[d], Tomaš Pilžys[c], Damian Garbicz[e], Naser Dib[f], Bianka Świderska[c], Piotr Matyba[a,] Zdzisław Gajewski[a], Elżbieta Grzesiuk[c*], Romuald Zabielski[a*]

[a] Center for Translational Medicine, Warsaw University of Life Sciences, Nowoursynowska 100, 02-797 Warsaw, Poland,

[b] Institute of Genetics and Biotechnology, Faculty of Biology, University of Warsaw, Pawińskiego 5a, 02-106 Warsaw, Poland,

[c] Institute of Biochemistry and Biophysics, Polish Academy of Sciences, Pawińskiego 5a, 02-106 Warsaw, Poland,

[d] Kielanowski Institute of Animal Physiology and Nutrition, Polish Academy of Sciences, Instytucka 3, 05-110 Jabłonna,

[e] Maria Sklodowska-Curie National Research, Institute of Oncology, W.K. Roentgena 5, 02-781 Warsaw, Poland,

[f] European Health Centre Otwock (ECZ Otwock), The Fryderyk Chopin Hospital, Borowa 14/18, 05-400 Otwock

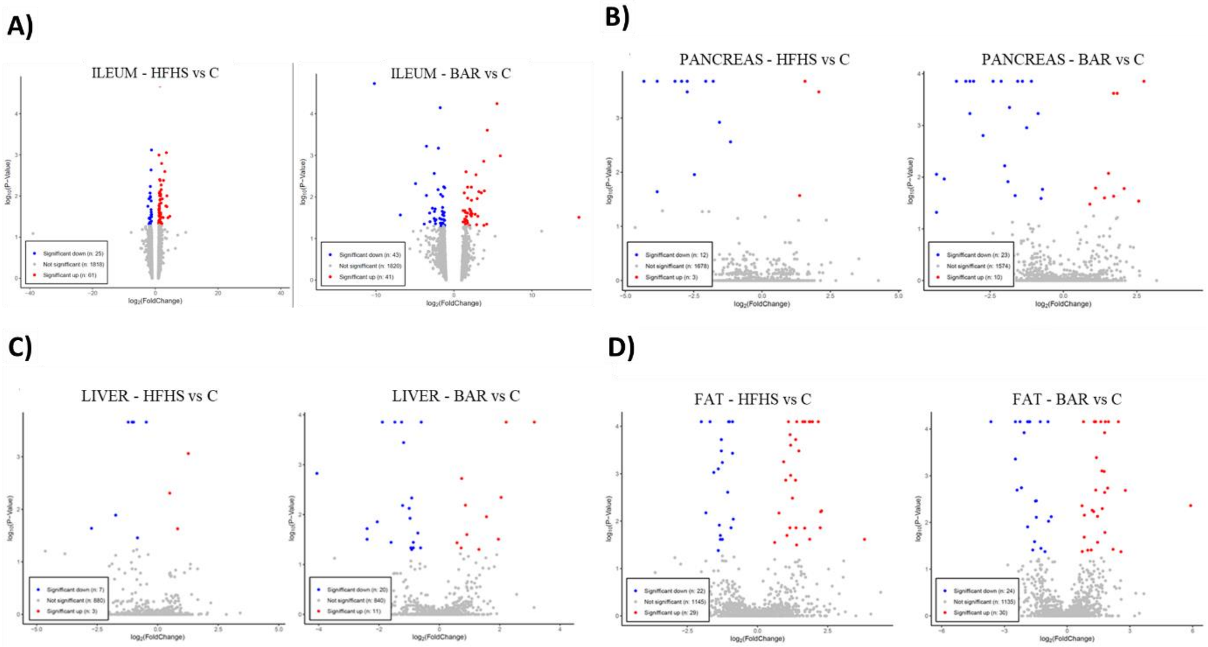

Suplementary Figure 1. **Evaluation of tissue specific proteome changes under conditions of high-energy diet or after bariatric surgery.** Data are presented as volcano plots of the entire set of proteins quantified during the proteomic analysis. Proteins, whose abundance was significantly changed are depicted in color. Blue dots indicate up-regulated proteins and red dots show down-regulated proteins in HFHS (left) and BAR (right), in comparison with the control. A) Ileum, B) Pancreas, C) Liver, D) Adipose Tissue.

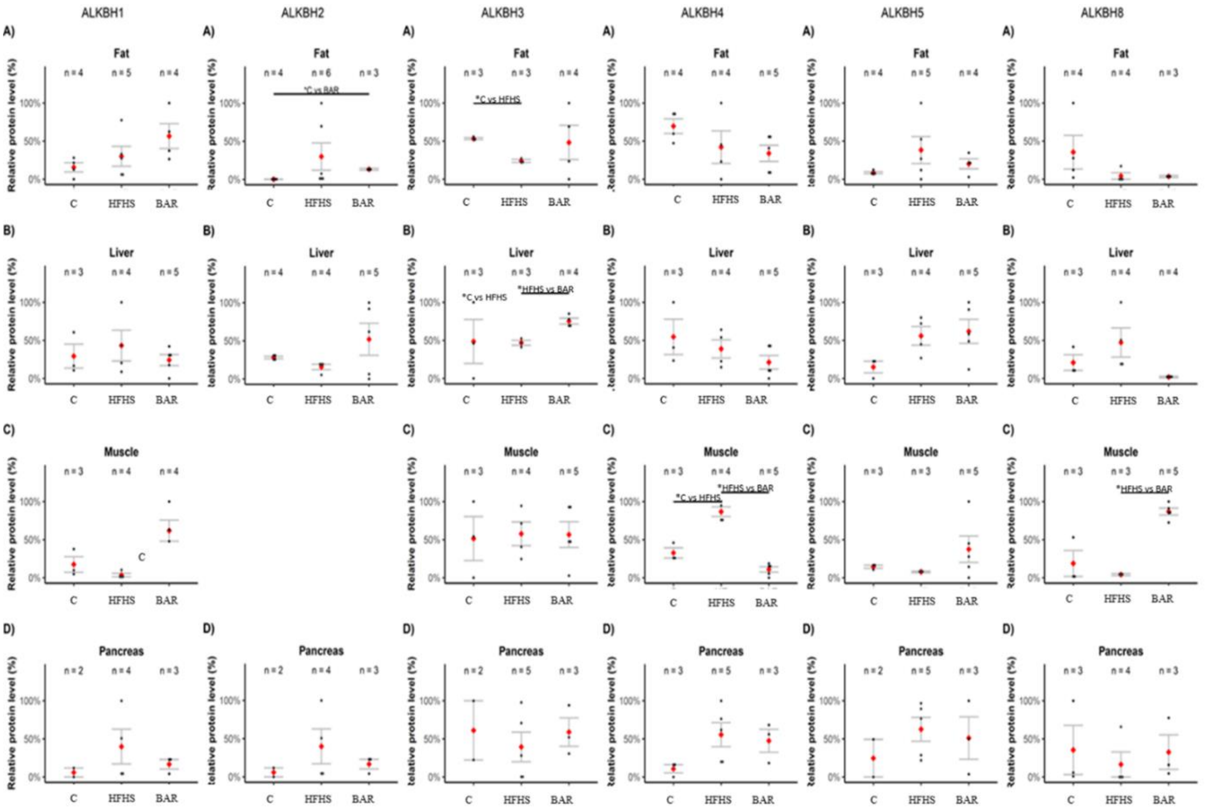

Suplementary Figure 2. **Post-western blot densitometry analysis of the ALKBH 1, 2, 3, 4, 5 and 8 protein level in selected pig tissue.** C - Standard diet (n = 6); HFHS - high energy diet with developed obesity and insulin resistance (n = 4); BAR - Pigs after bariatric surgery (n=6). Data are represented as points and means (red), with the whiskers (grey) showing sample standard deviation. n - number of samples. *$p < 0.05$, two-tailed unpaired Student's *t* test with Benjamini-Hochberg adjustment (software R version 3.3.0).

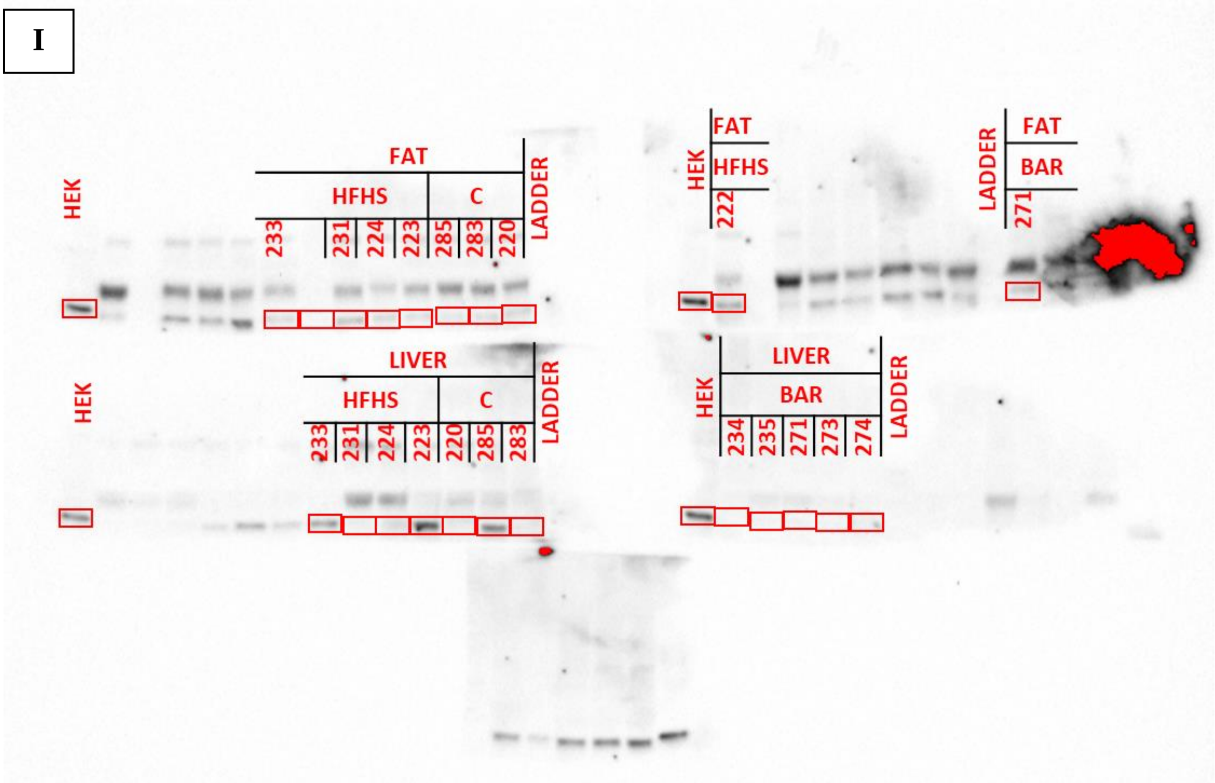
I
HEK
FAT
HFHS
C
233
231
224
223
285
283
220
LADDER
HEK
FAT
HFHS
222
LADDER
FAT
BAR
271
HEK
LIVER
HFHS
C
233
231
224
223
220
285
283
LADDER
HEK
LIVER
BAR
234
235
271
273
274
LADDER

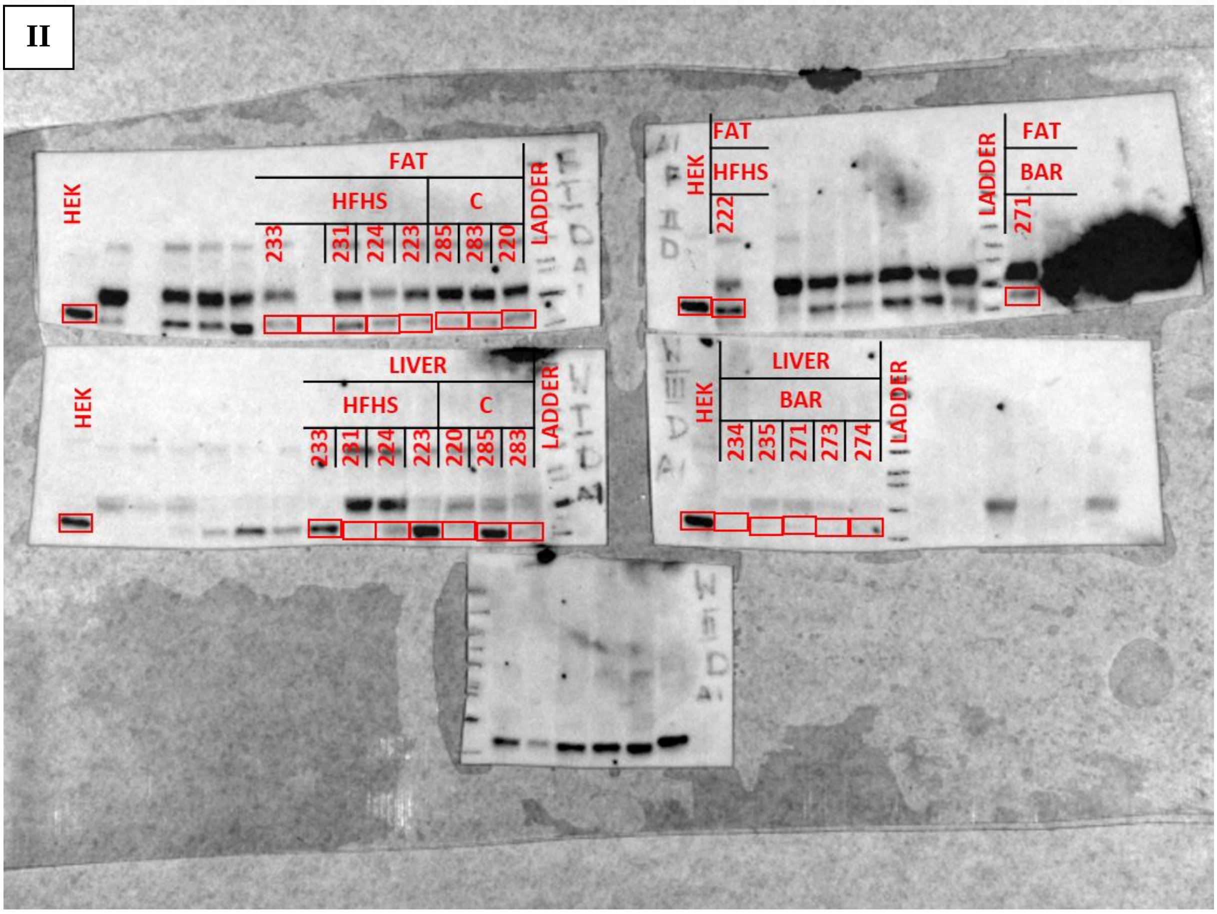
II
HEK
FAT
HFHS
C
233
231
224
223
285
283
220
LADDER
HEK
FAT
HFHS
222
LADDER
FAT
BAR
271
HEK
LIVER
HFHS
C
233
231
224
223
220
285
283
LADDER
HEK
LIVER
BAR
234
235
271
273
274
LADDER

III

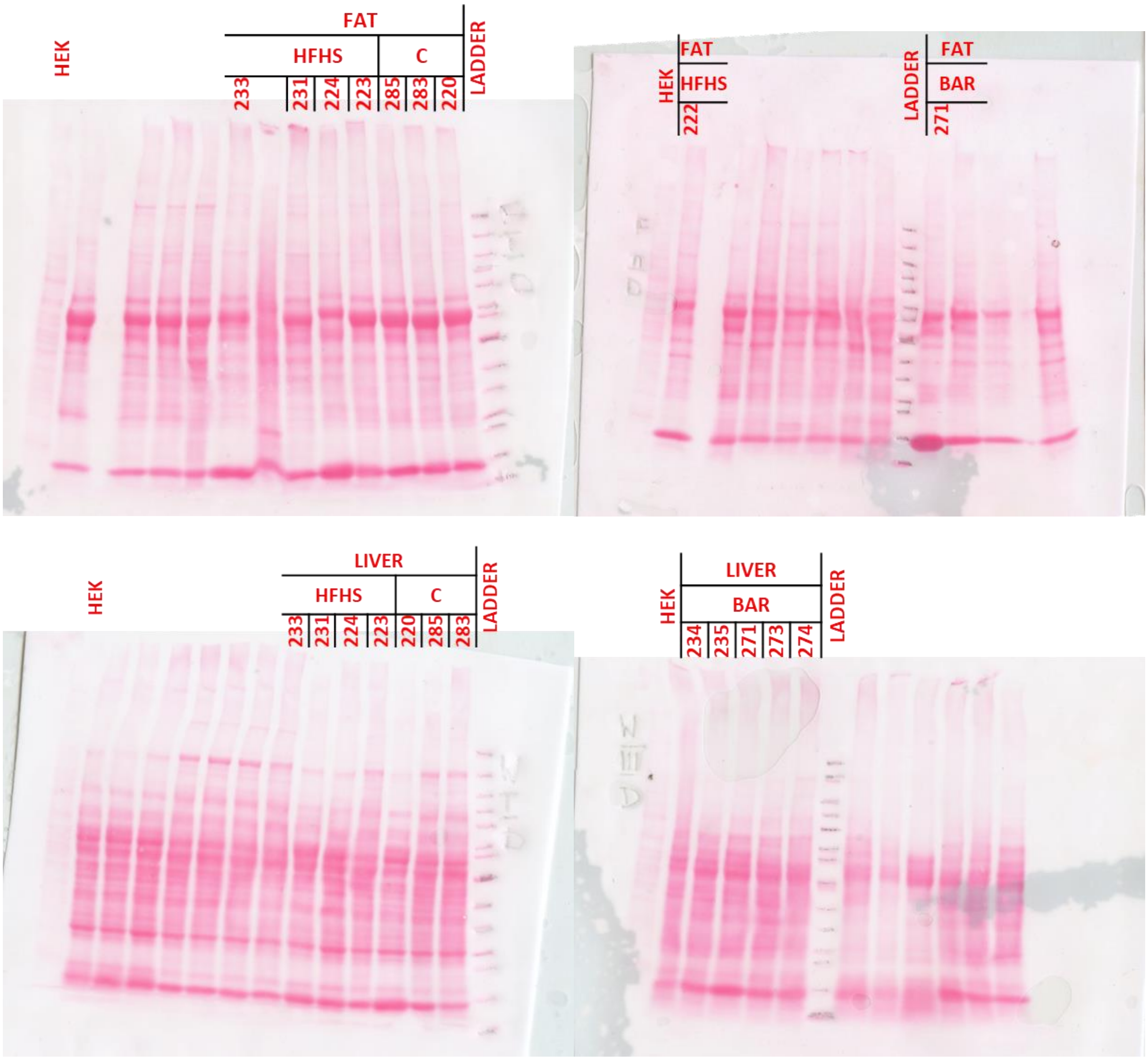
HEK
FAT
HFHS
C
LADDER
233
231
224
223
285
283
220
FAT
HEK
HFHS
222
LADDER
FAT
BAR
271
LIVER
HEK
HFHS
C
LADDER
233
231
224
223
220
285
283
LIVER
HEK
BAR
234
235
271
273
274
LADDER

I

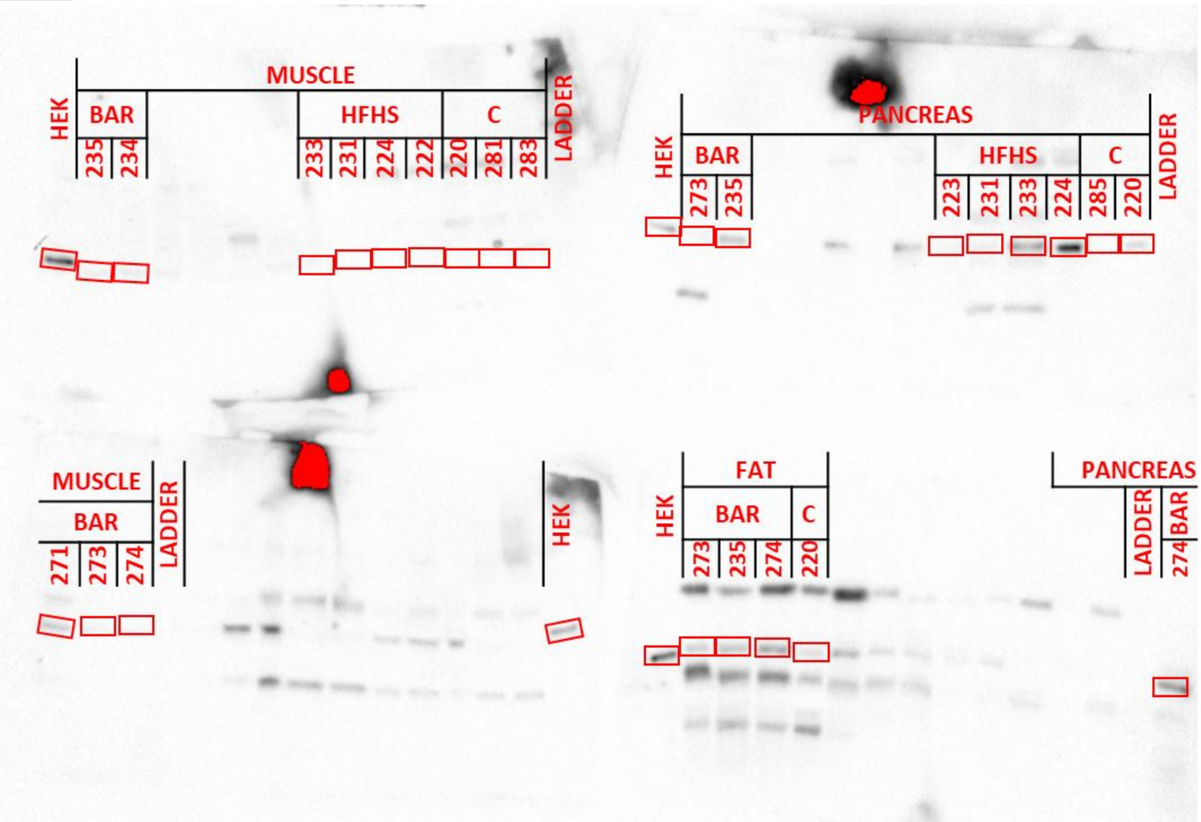
MUSCLE
HEK
BAR
235 234
HFHS
233 231 224 222
C
220 281 283
LADDER
PANCREAS
HEK
BAR
273 235
HFHS
223 231 233 224
C
285 220
LADDER
MUSCLE
BAR
271 273 274
LADDER
HEK
FAT
HEK
BAR
273 235 274
C
220
PANCREAS
LADDER
BAR
274

II

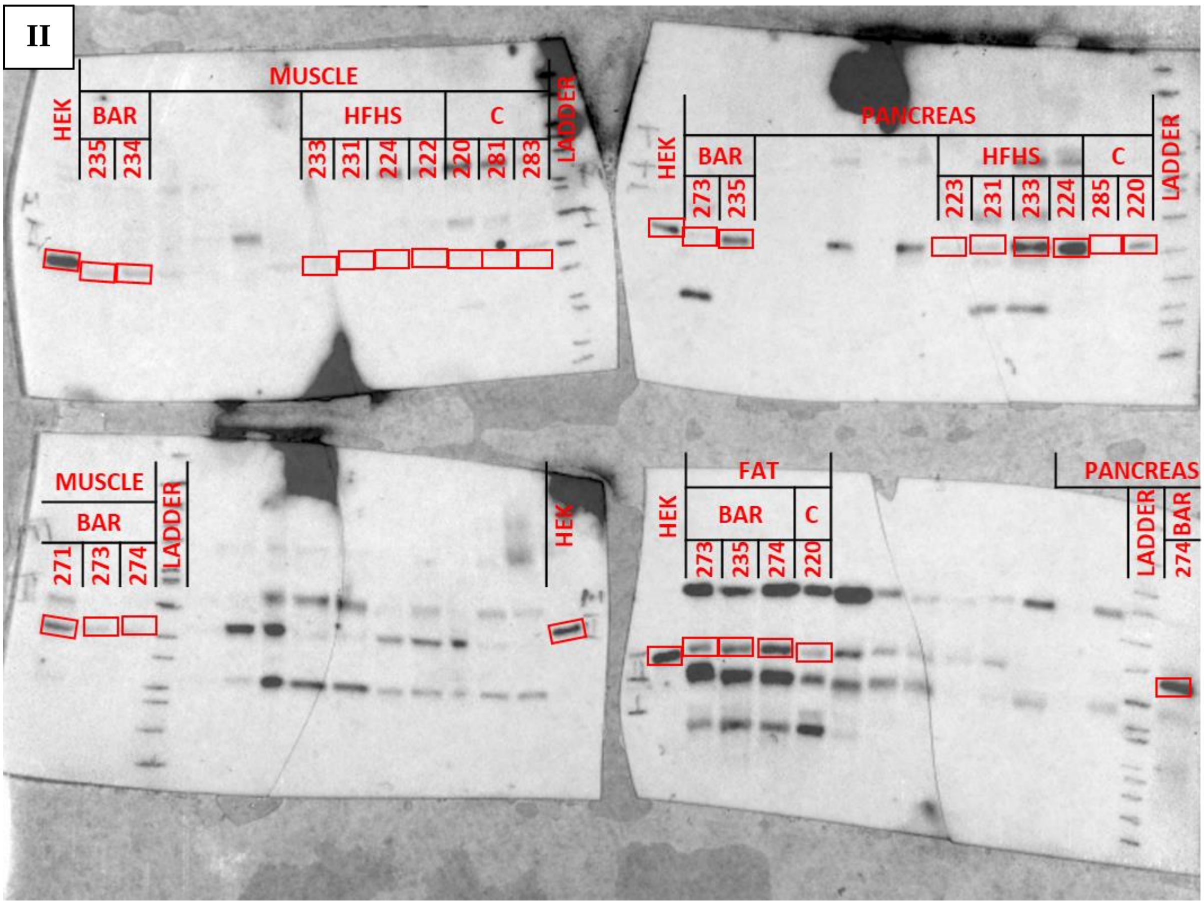
MUSCLE
HEK
BAR
235 234
HFHS
233 231 224 222
C
220 281 283
LADDER
PANCREAS
HEK
BAR
273 235
HFHS
223 231 233 224
C
285 220
LADDER
MUSCLE
BAR
271 273 274
LADDER
HEK
FAT
HEK
BAR
273 235 274
C
220
PANCREAS
LADDER
BAR
274

III

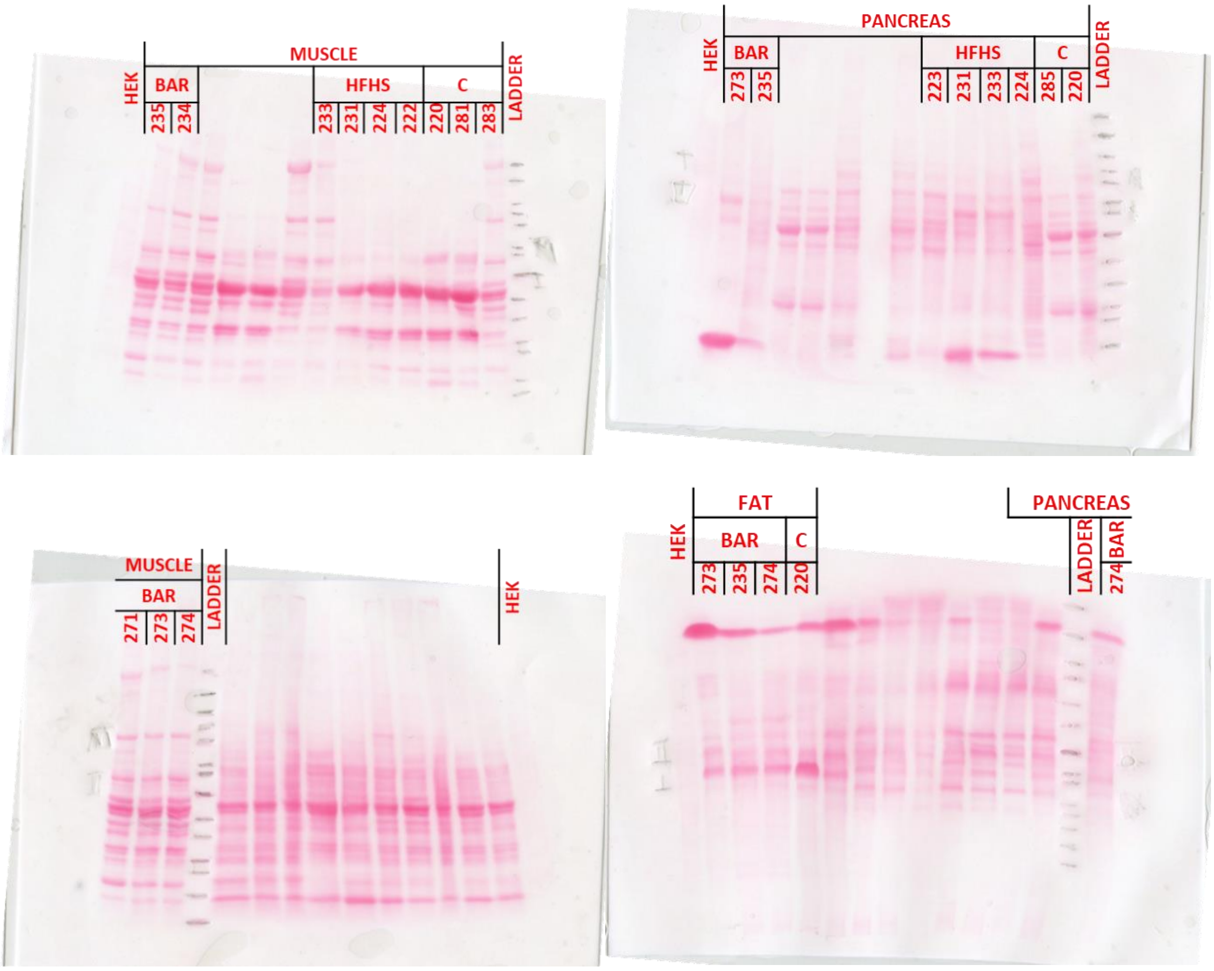

Suplementary Figure 3. **Western blot analysis of ALKBH1 protein.** The bars selected for analysis are marked with red squares. The overexposed signal is highlighted in red. The amount of signal from the sample was normalised by dividing the value by the amount of signal from the HEK293 cell line (HEK) sample. Invastigated groups: C - Standard diet (n = 6); HFHS - high energy diet with developed obesity and insulin resistance (n = 4); BAR - Pigs after bariatric surgery (n=6); Investigated tissues: M – muscle; P – pancreas; L – Liver; F – Adipose tissue. I – Image of exposition from which densitometric data were obtained; II – Overview image of long exposition showing the blots including their framing, possible cutting locations and molecular ladder markings. III – Overview image of the blots after Ponceu Red stayning, for total protein verification after protein transfer on the membrane.

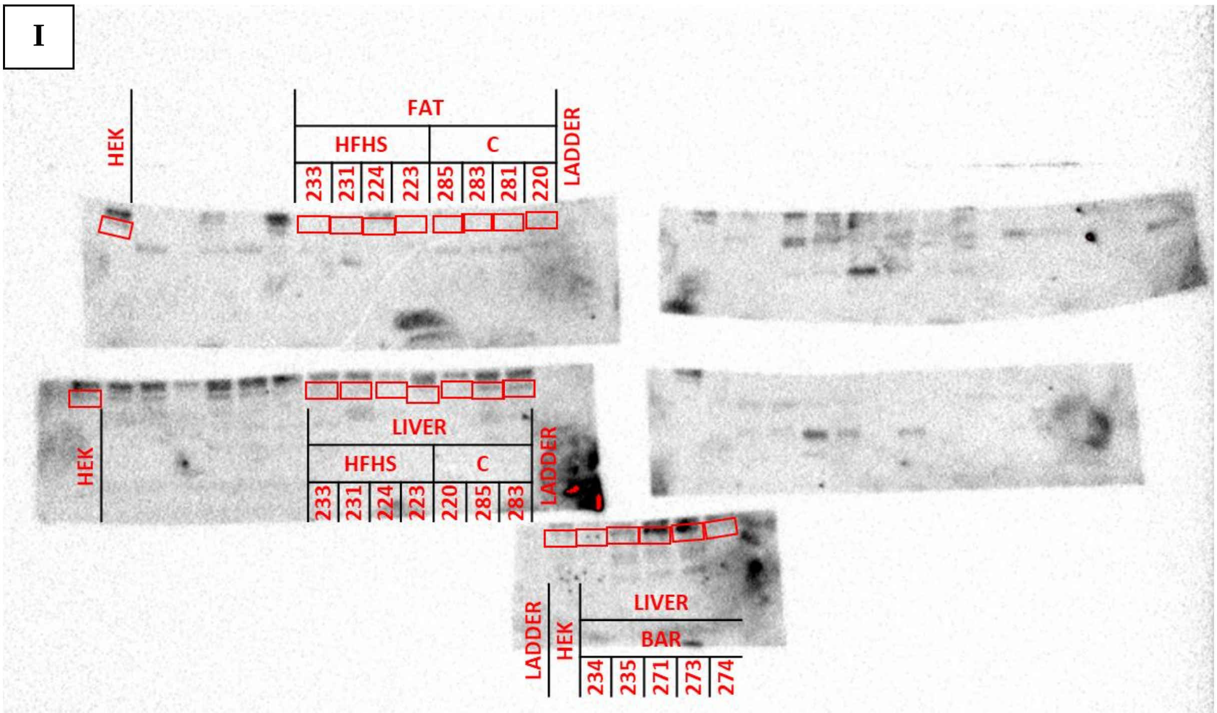
I
HEK
FAT
HFHS
C
233
231
224
223
285
283
281
220
LADDER
LIVER
HFHS
C
233
231
224
223
220
285
283
LADDER
LIVER
BAR
LADDER
HEK
234
235
271
273
274

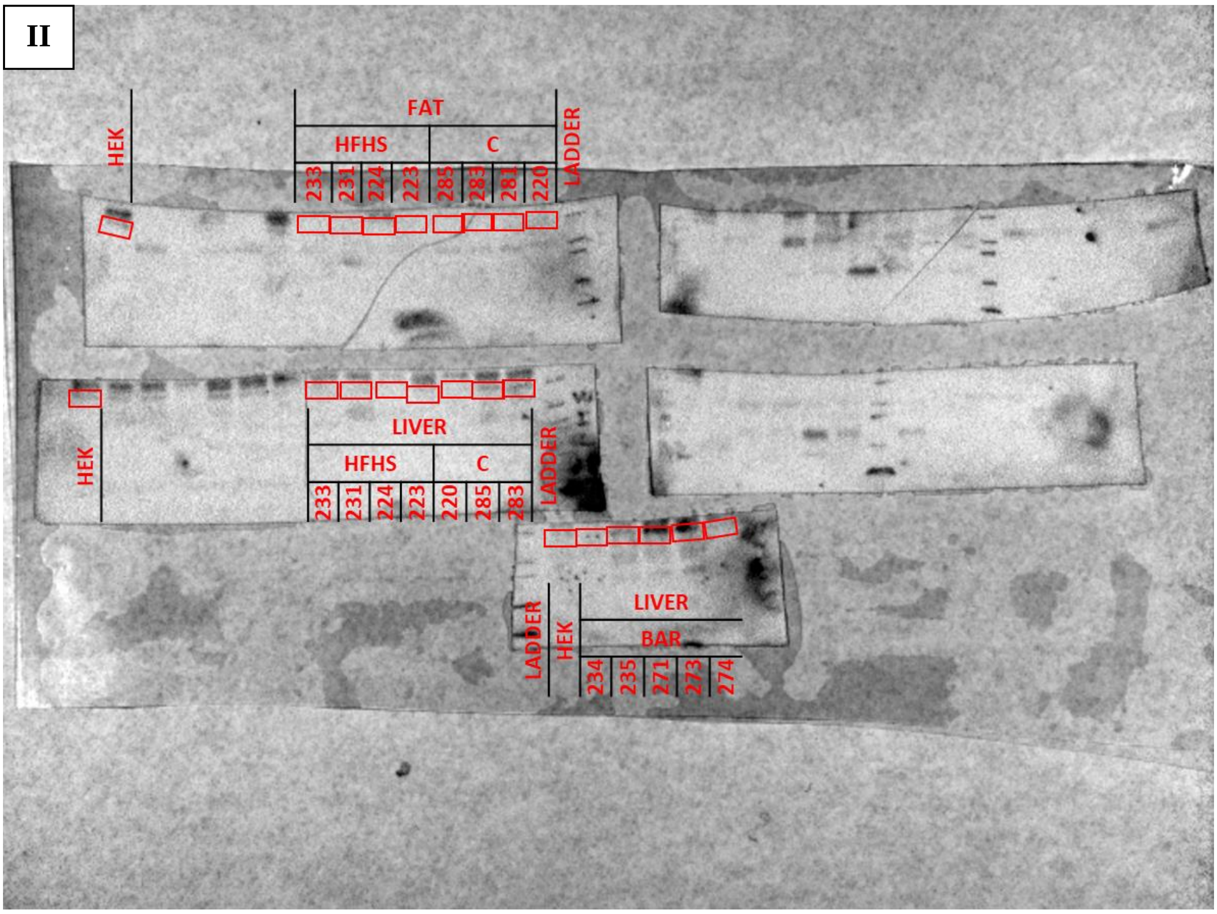
II
HEK
FAT
HFHS
C
233
231
224
223
285
283
281
220
LADDER
LIVER
HFHS
C
233
231
224
223
220
285
283
LADDER
LIVER
BAR
LADDER
HEK
234
235
271
273
274

III

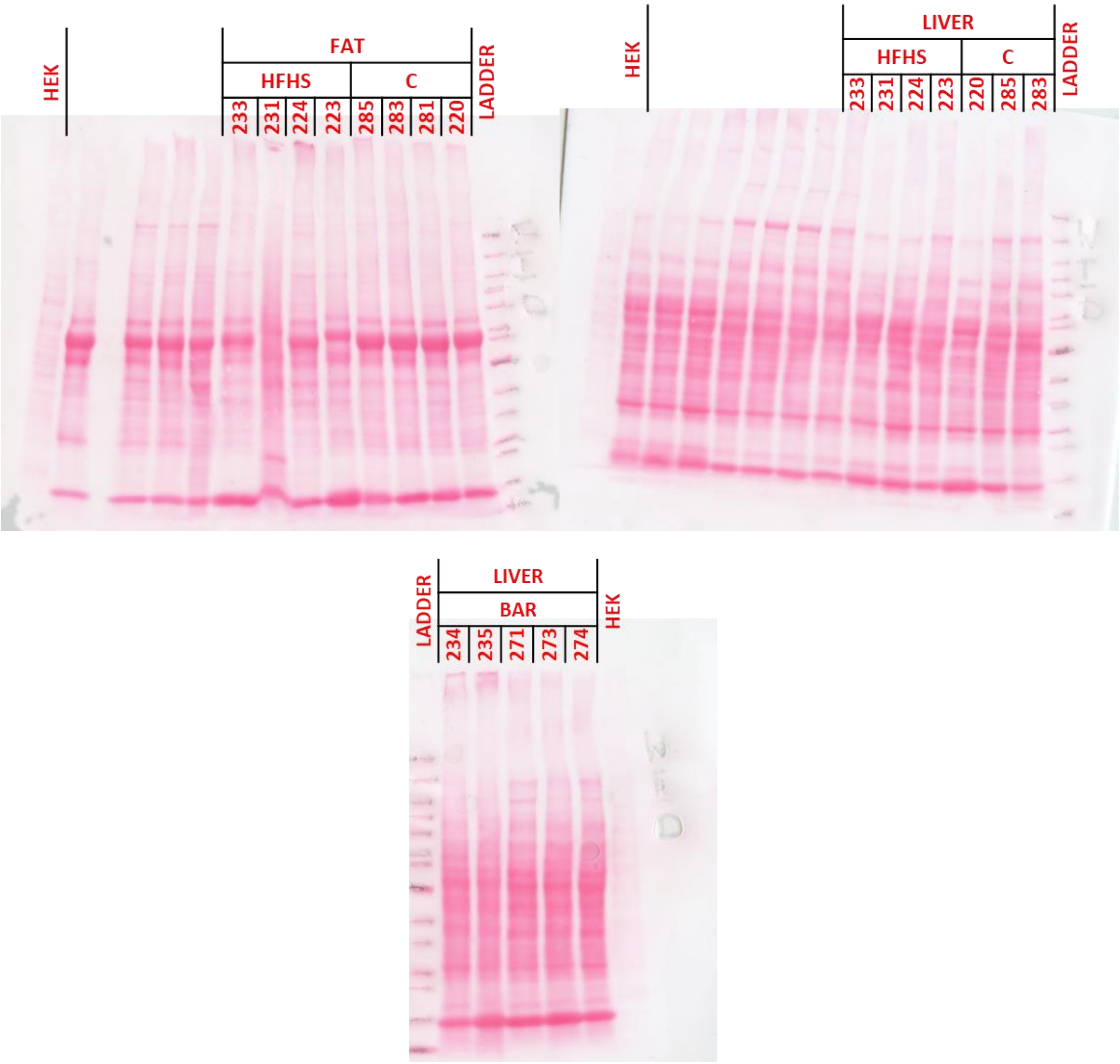
HEK
FAT
HFHS
C
233
231
224
223
285
283
281
220
LADDER
HEK
LIVER
HFHS
C
233
231
224
223
220
285
283
LADDER
LADDER
LIVER
BAR
234
235
271
273
274
HEK

I

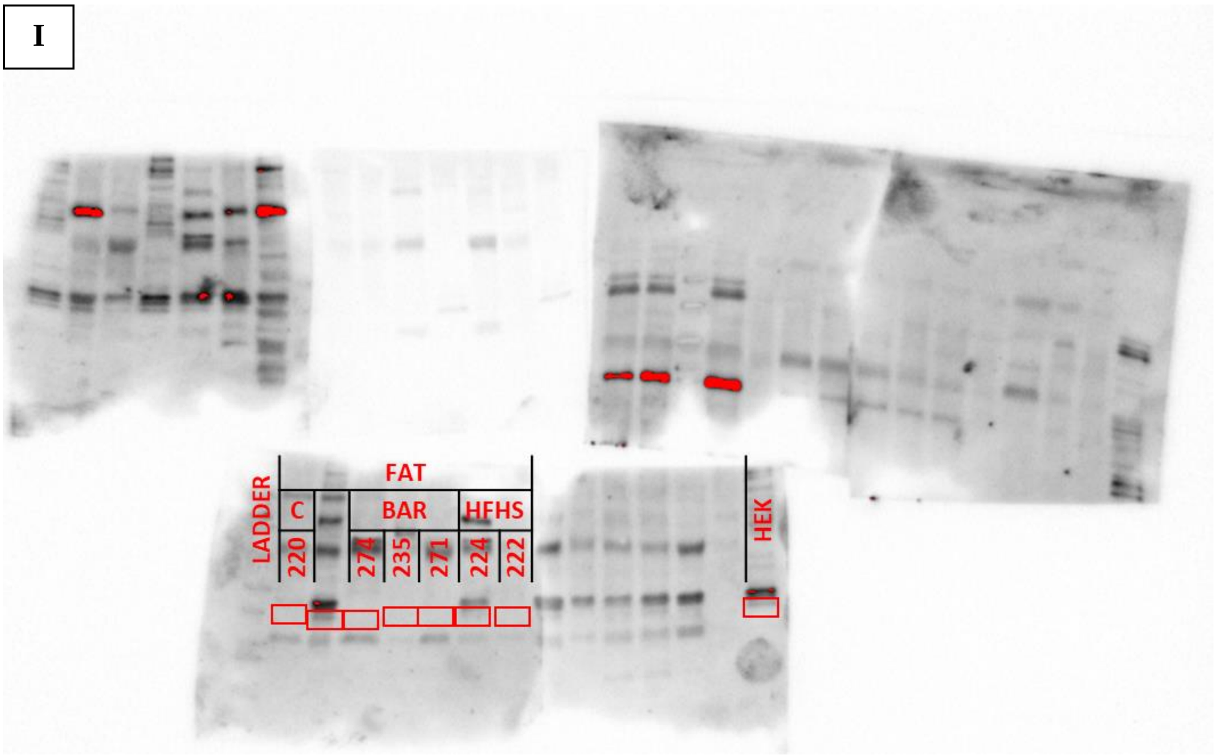

LADDER
FAT
C
BAR
HFHS
220
274
235
271
224
222
HEK

II

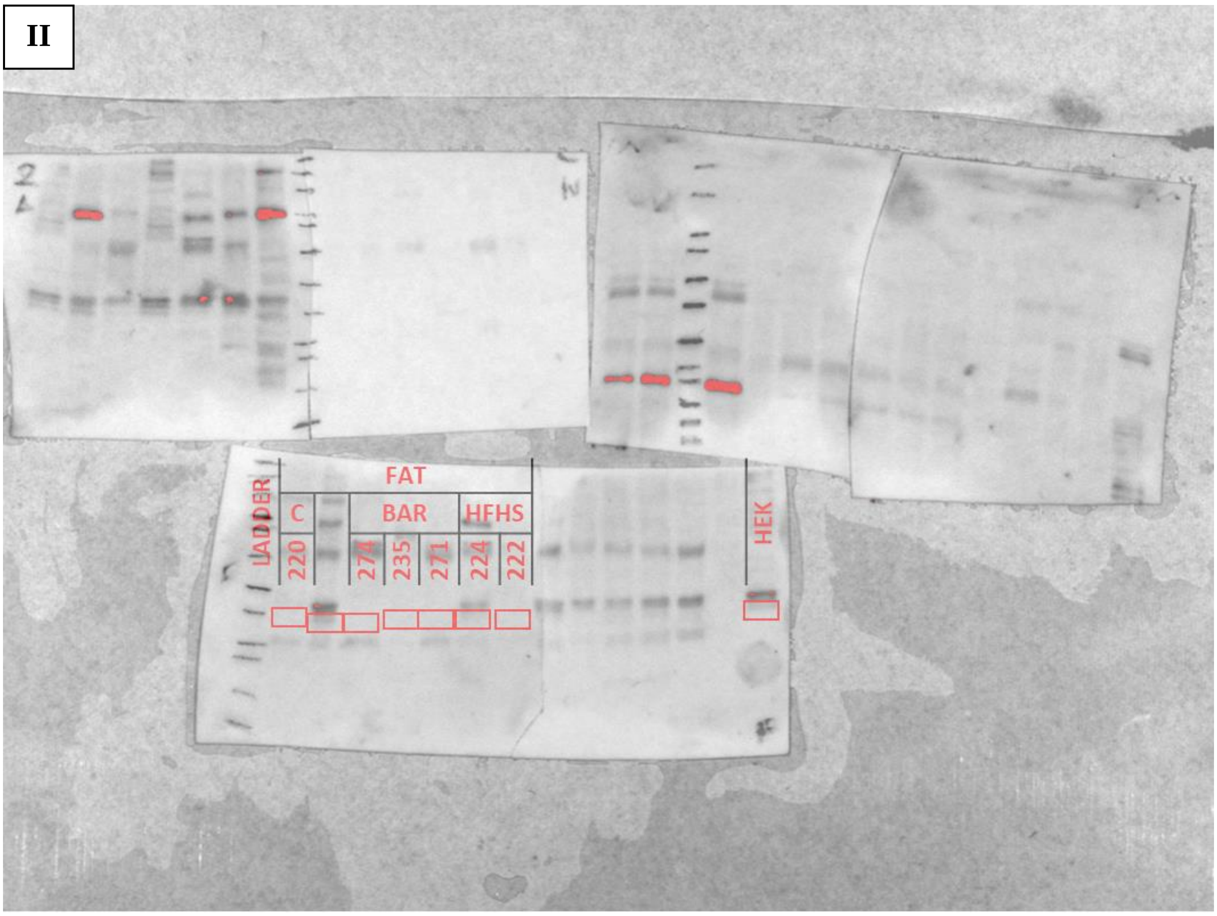

LADDER
FAT
C
BAR
HFHS
220
274
235
271
224
222
HEK

III

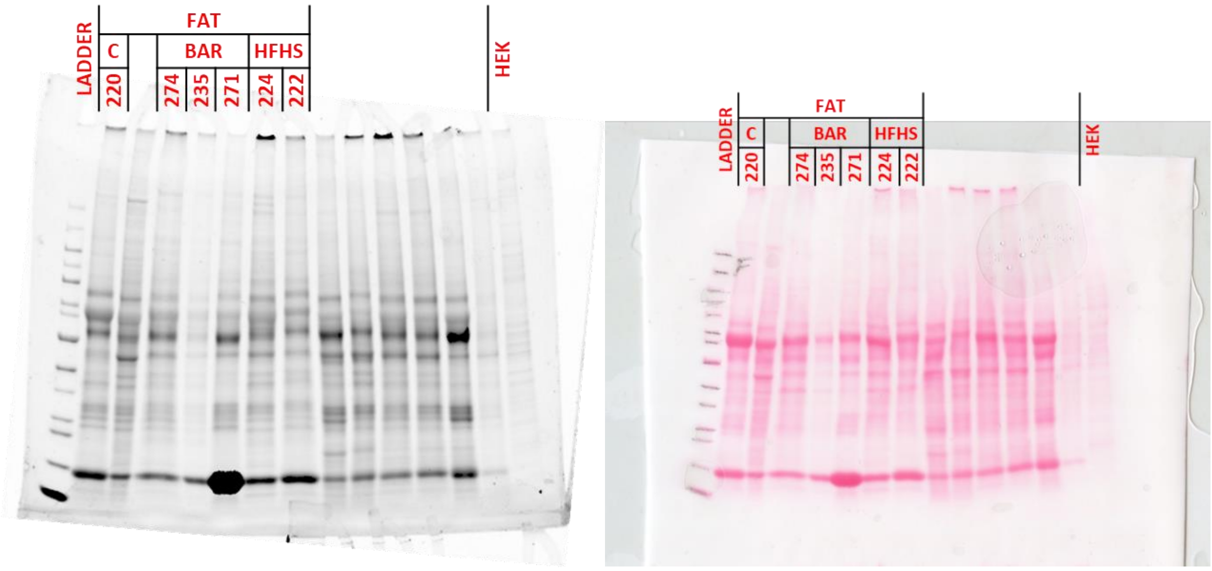

Suplementary Figure 4. **Western blot analysis of ALKBH2 protein.** The bars selected for analysis are marked with red squares. The overexposed signal is highlighted in red. The amount of signal from the sample was normalised by dividing the value by the amount of signal from the HEK293 cell line (HEK) sample. Invastigated groups: C - Standard diet (n = 6); HFHS - high energy diet with developed obesity and insulin resistance (n = 4); BAR - Pigs after bariatric surgery (n=6); Invastigated tissues: M – muscle; P – pancreas; L – Liver; F – Adipose tissue. I – Image of exposition from which densitometric data were obtained; II – Overview image of long exposition showing the blots including their framing, possible cutting locations and molecular ladder markings. III – Overview image of the visualized Stein-free gel and the blot after Ponceau Red staining, for total protein verification after protein transfer on the membrane.

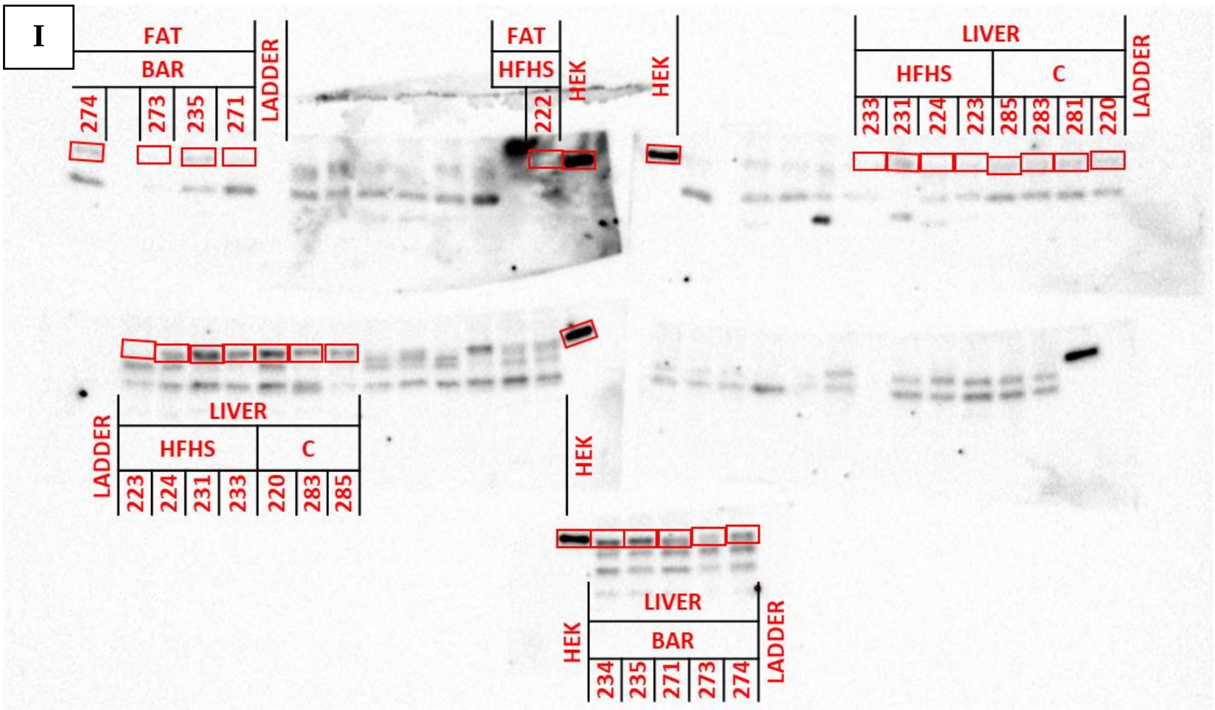

II

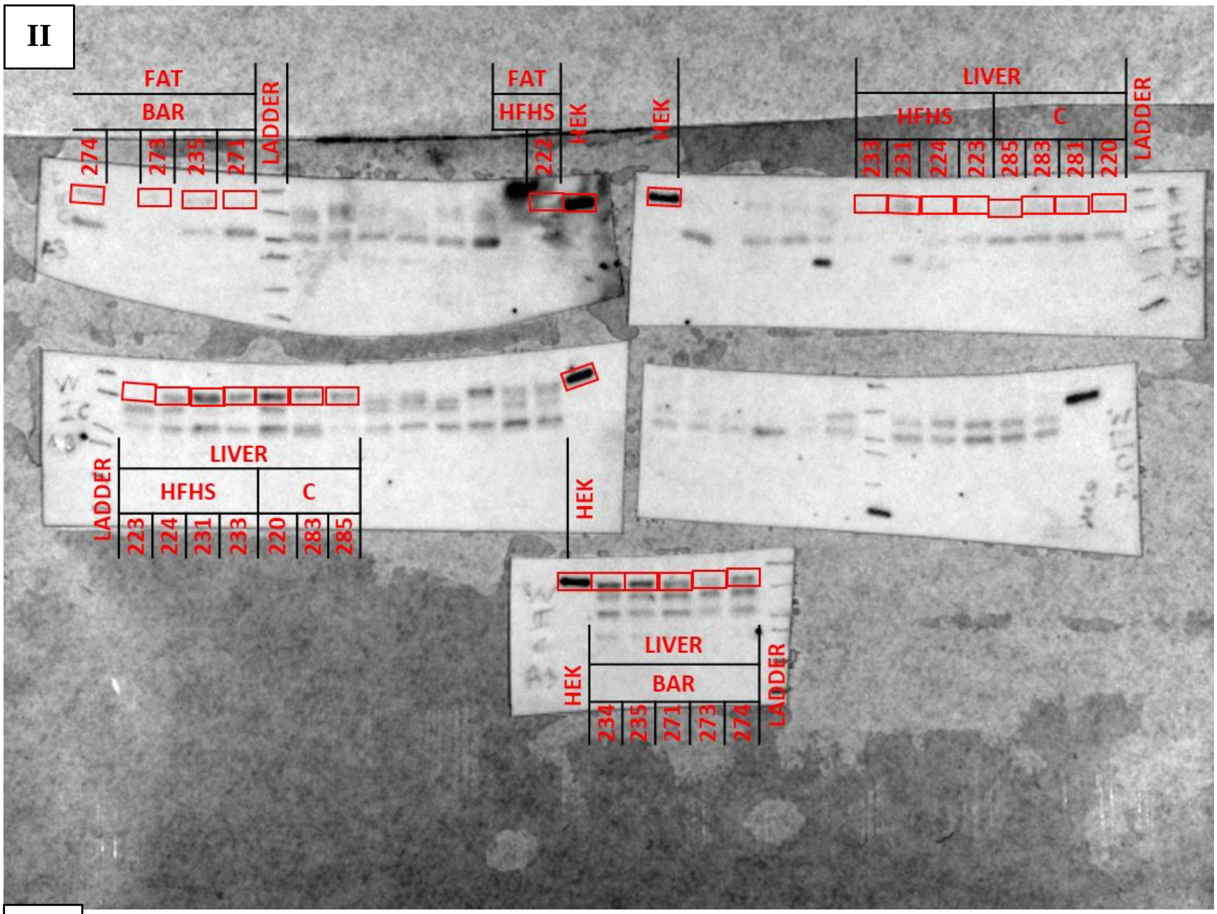
FAT
BAR
274
273
235
271
LADDER
FAT
HFHS
222
HEK
HEK
LIVER
HFHS
C
233
231
224
223
285
283
281
220
LADDER
LIVER
HFHS
C
LADDER
223
224
231
233
220
283
285
HEK
LIVER
BAR
HEK
234
235
271
273
274
LADDER

III

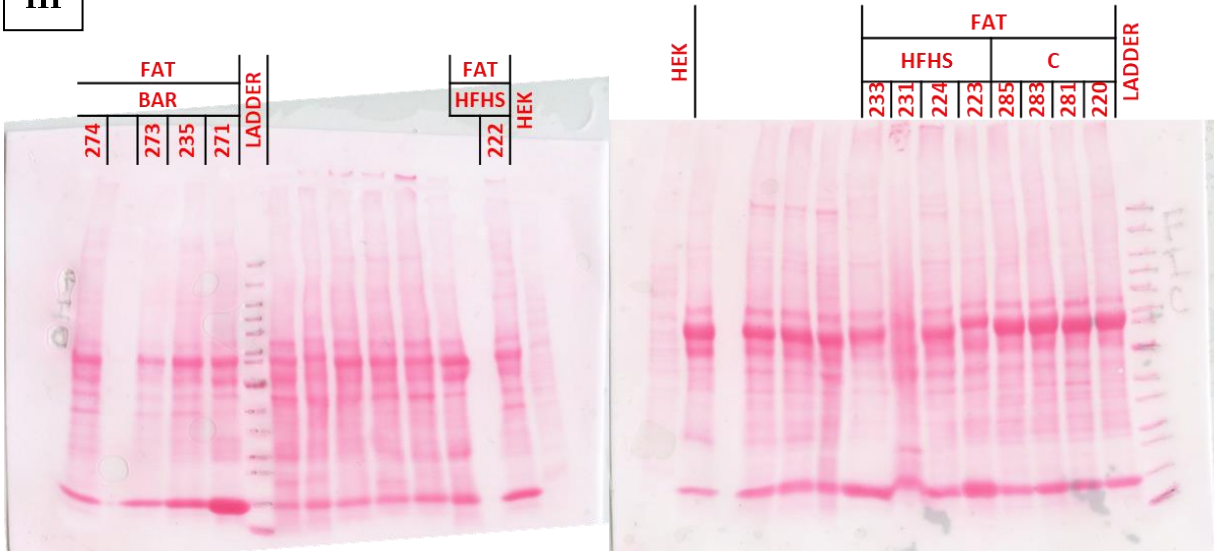
FAT
BAR
274
273
235
271
LADDER
FAT
HFHS
222
HEK
HEK
FAT
HFHS
C
233
231
224
223
285
283
281
220
LADDER

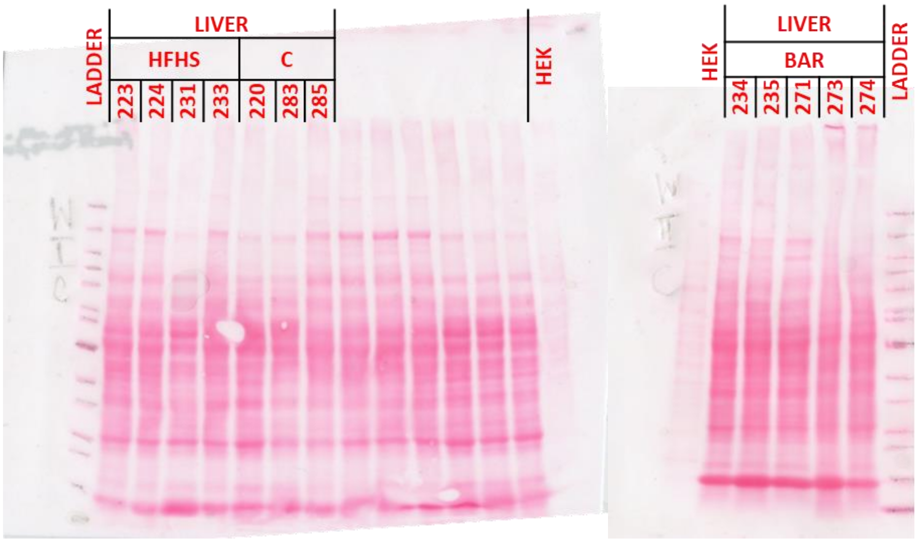
LADDER
LIVER
HFHS
C
223
224
231
233
220
283
285
HEK
HEK
LIVER
BAR
234
235
271
273
274
LADDER

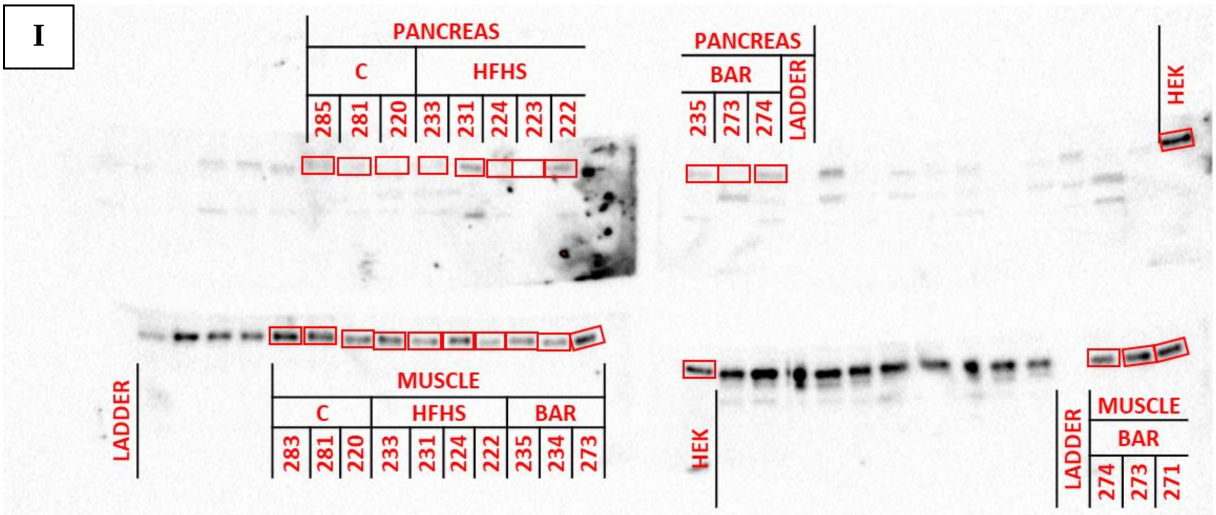
I
PANCREAS
C
HFHS
285
281
220
233
231
224
223
222
PANCREAS
BAR
235
273
274
LADDER
HEK
LADDER
MUSCLE
C
HFHS
BAR
283
281
220
233
231
224
222
235
234
273
HEK
LADDER
MUSCLE
BAR
274
273
271

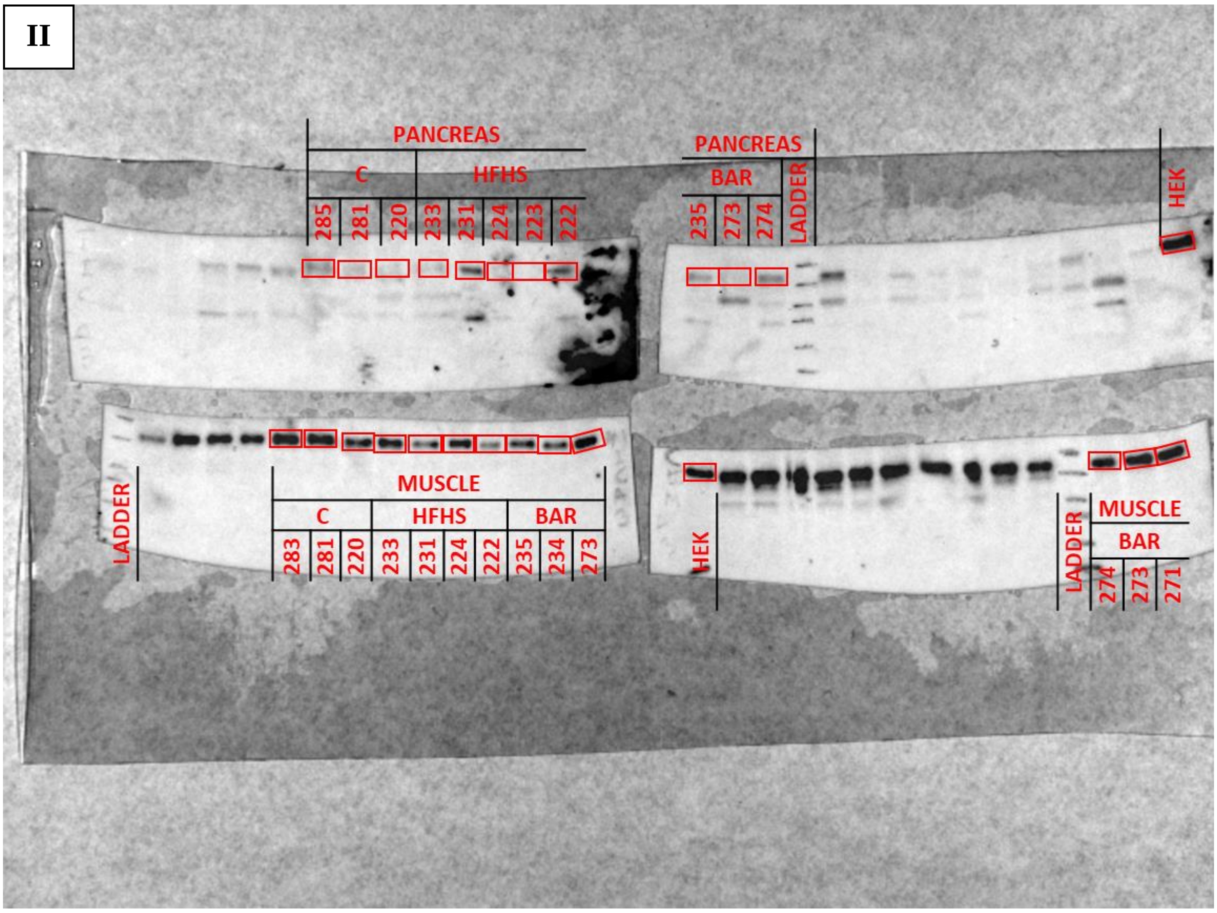
II
PANCREAS
C
HFHS
285
281
220
233
231
224
223
222
PANCREAS
BAR
235
273
274
LADDER
HEK
LADDER
MUSCLE
C
HFHS
BAR
283
281
220
233
231
224
222
235
234
273
HEK
LADDER
MUSCLE
BAR
274
273
271

III

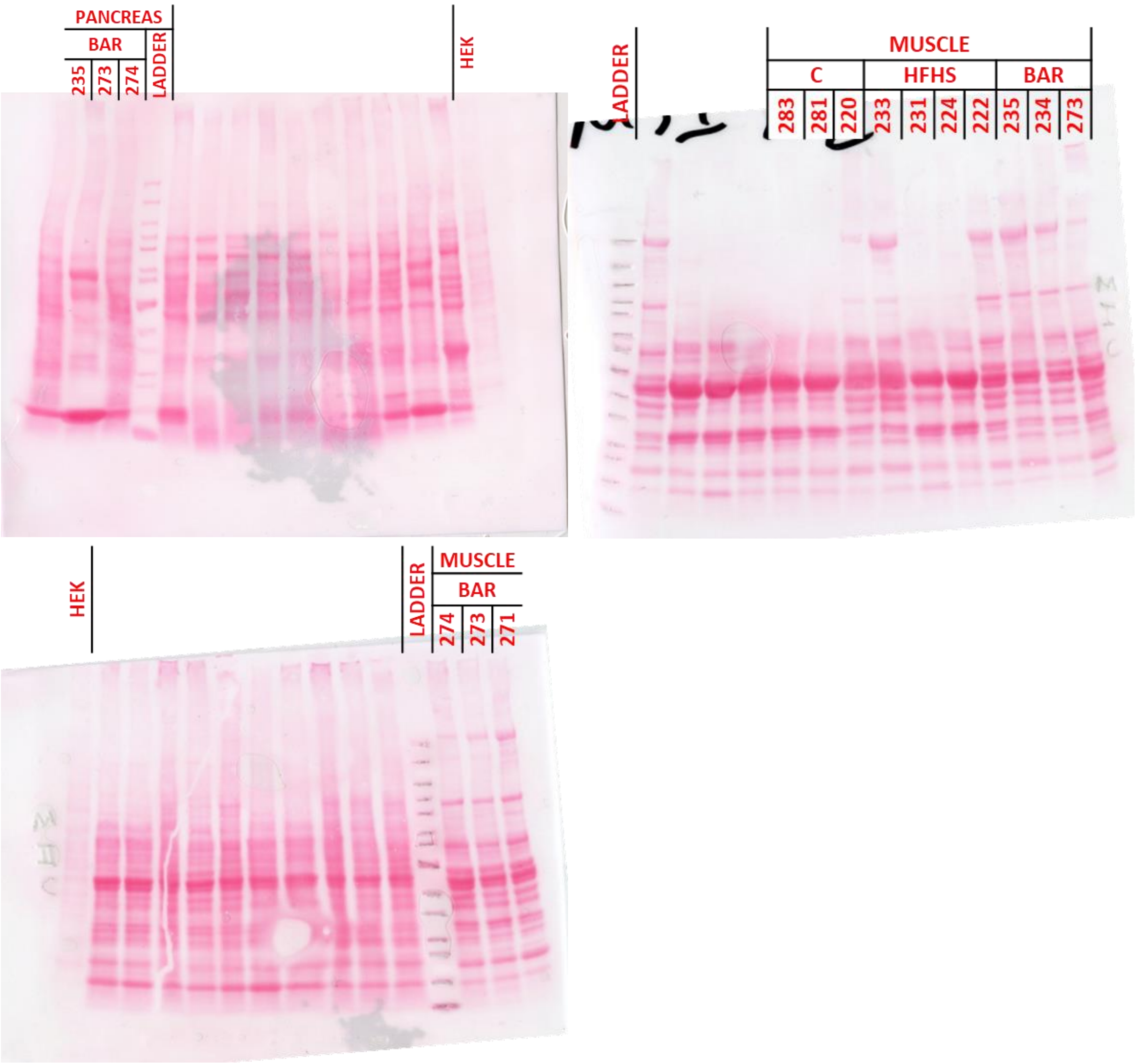

Suplementary Figure 5. **Western blot analysis of ALKBH3 protein.** The bars selected for analysis are marked with red squares. The overexposed signal is highlighted in red. The amount of signal from the sample was normalised by dividing the value by the amount of signal from the HEK293 cell line (HEK) sample. Invastigated groups: C - Standard diet (n = 6); HFHS - high energy diet with developed obesity and insulin resistance (n = 4); BAR - Pigs after bariatric surgery (n=6); Invastigated tissues: M – muscle; P – pancreas; L – Liver; F – Adipose tissue. I – Image of exposition from which densitometric data were obtained; II – Overview image of long exposition showing the blots including their framing, possible cutting locations and molecular ladder markings. III – Overview image of the blots after Ponceu Red stayning, for total protein verification after protein transfer on the membrane.

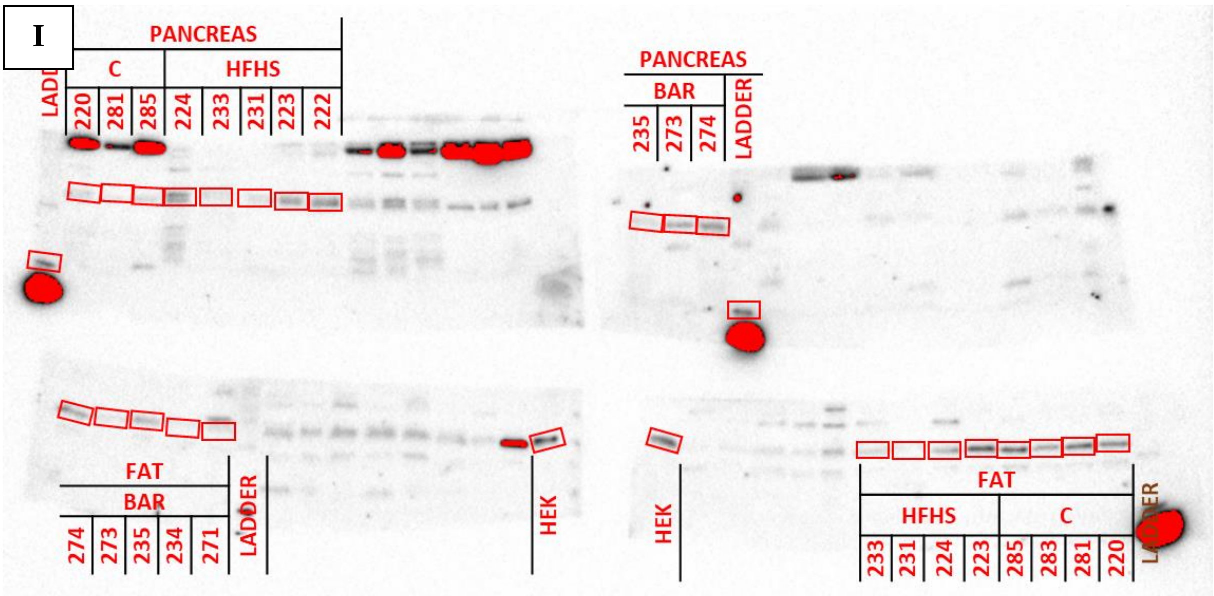
I
PANCREAS
C
HFHS
LADD
220
281
285
224
233
231
223
222
PANCREAS
BAR
235
273
274
LADDER
FAT
BAR
274
273
235
234
271
LADDER
HEK
HEK
FAT
HFHS
C
233
231
224
223
285
283
281
220
LADDER

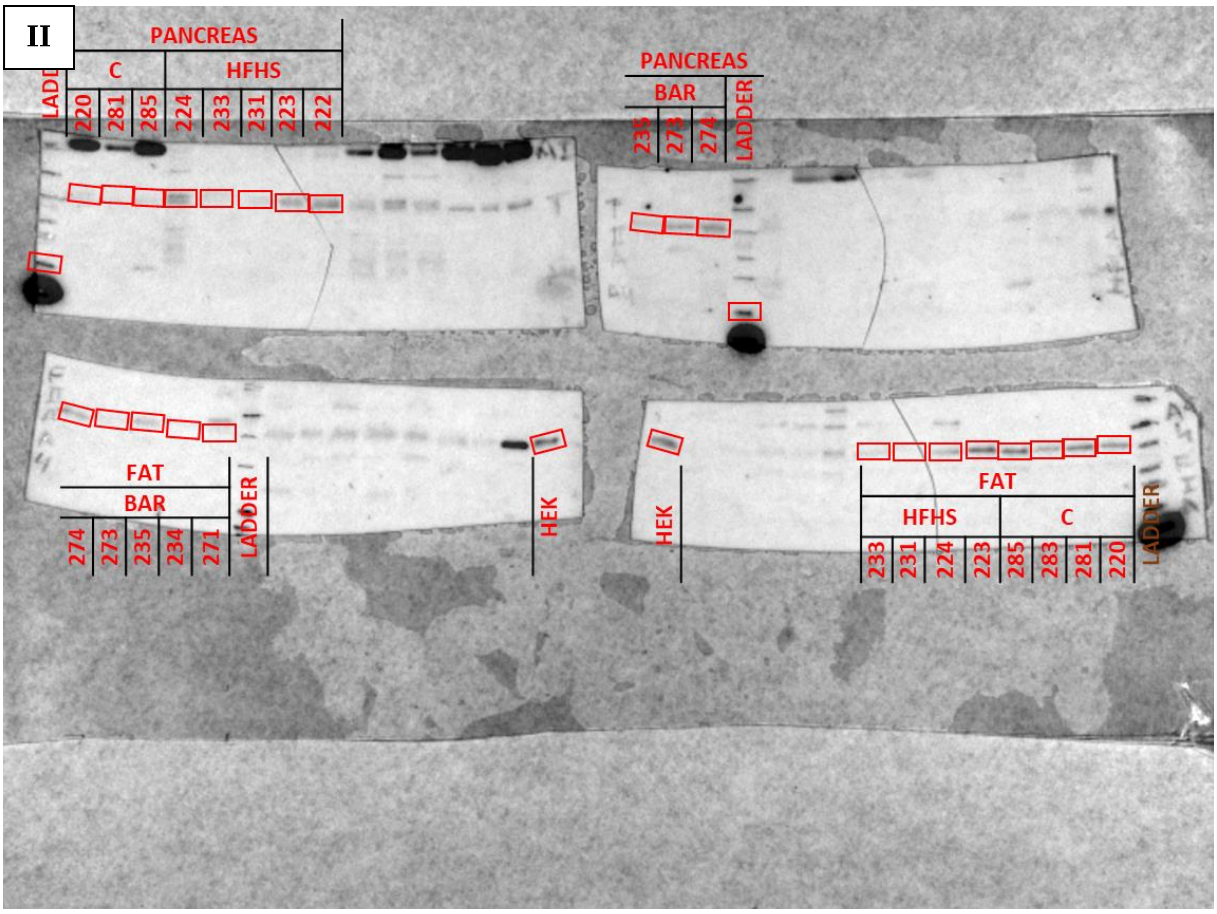
II
PANCREAS
C
HFHS
LADD
220
281
285
224
233
231
223
222
PANCREAS
BAR
235
273
274
LADDER
FAT
BAR
274
273
235
234
271
LADDER
HEK
HEK
FAT
HFHS
C
233
231
224
223
285
283
281
220
LADDER

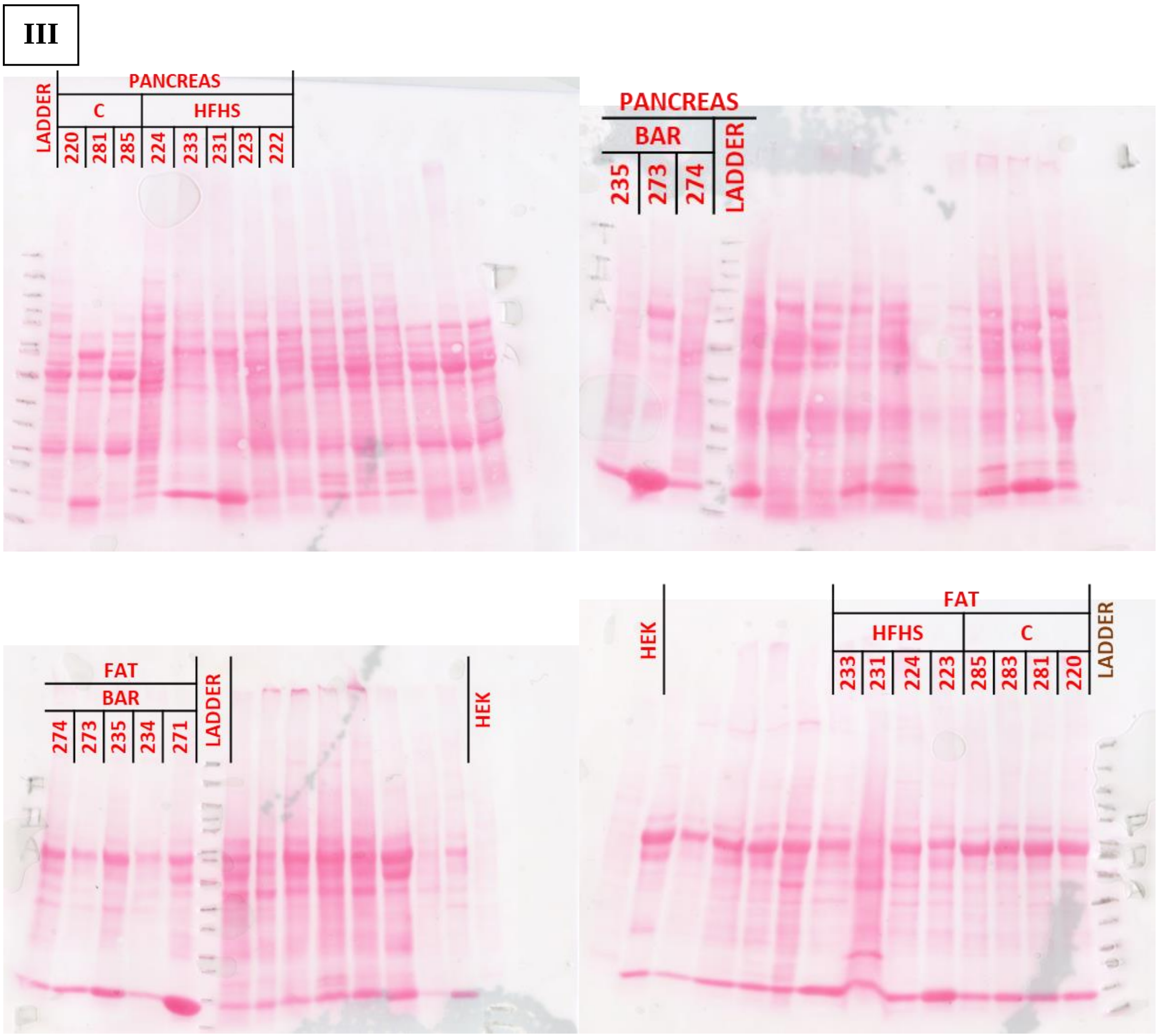
III
LADDER
PANCREAS
C
HFHS
220
281
285
224
233
231
223
222
PANCREAS
BAR
235
273
274
LADDER
FAT
BAR
274
273
235
234
271
LADDER
HEK
HEK
FAT
HFHS
C
233
231
224
223
285
283
281
220
LADDER

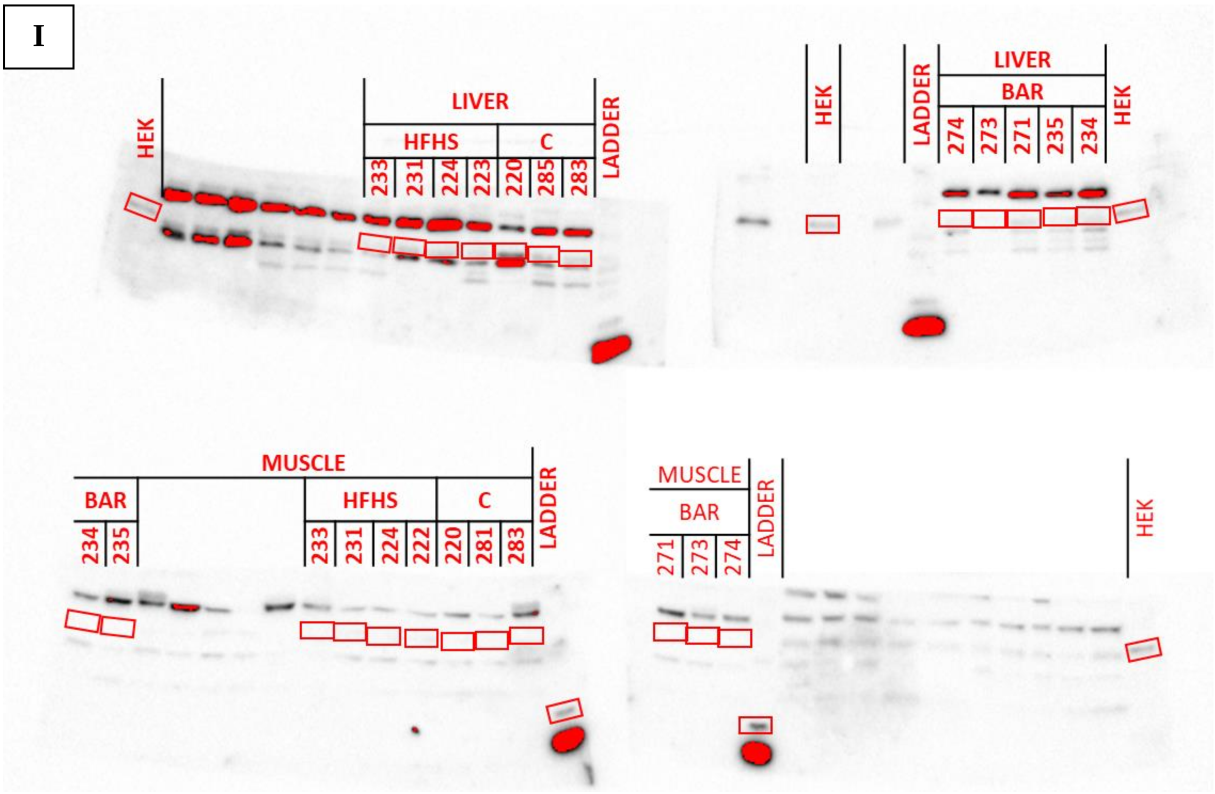
I
HEK
LIVER
HFHS
C
233
231
224
223
220
285
283
LADDER
HEK
LADDER
LIVER
BAR
274
273
271
235
234
HEK
MUSCLE
BAR
234
235
HFHS
C
233
231
224
222
220
281
283
LADDER
MUSCLE
BAR
271
273
274
LADDER
HEK

II

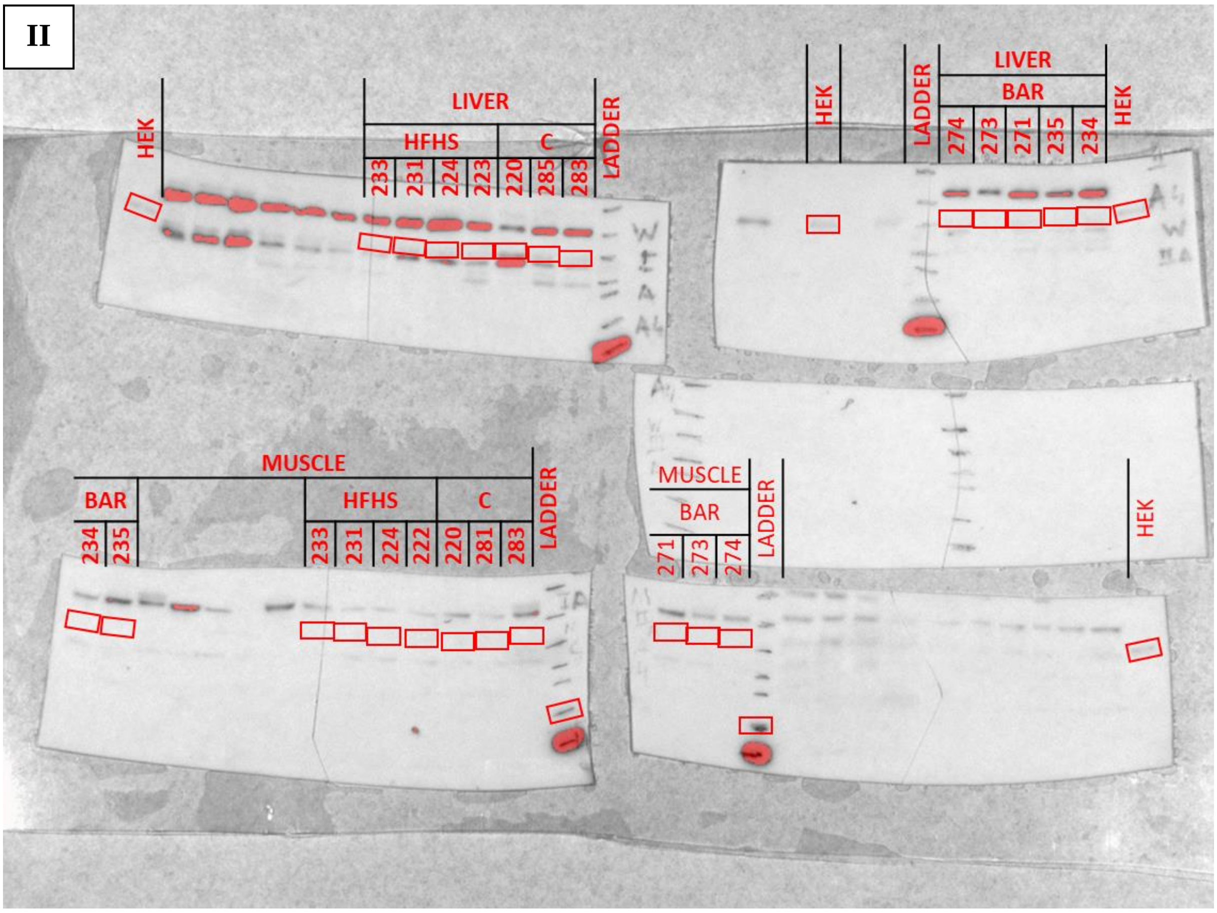
HEK
LIVER
HFHS
C
233
231
224
223
220
285
283
LADDER
HEK
LADDER
LIVER
BAR
274
273
271
235
234
HEK
MUSCLE
BAR
234
235
HFHS
C
233
231
224
222
220
281
283
LADDER
MUSCLE
BAR
271
273
274
LADDER
HEK

III

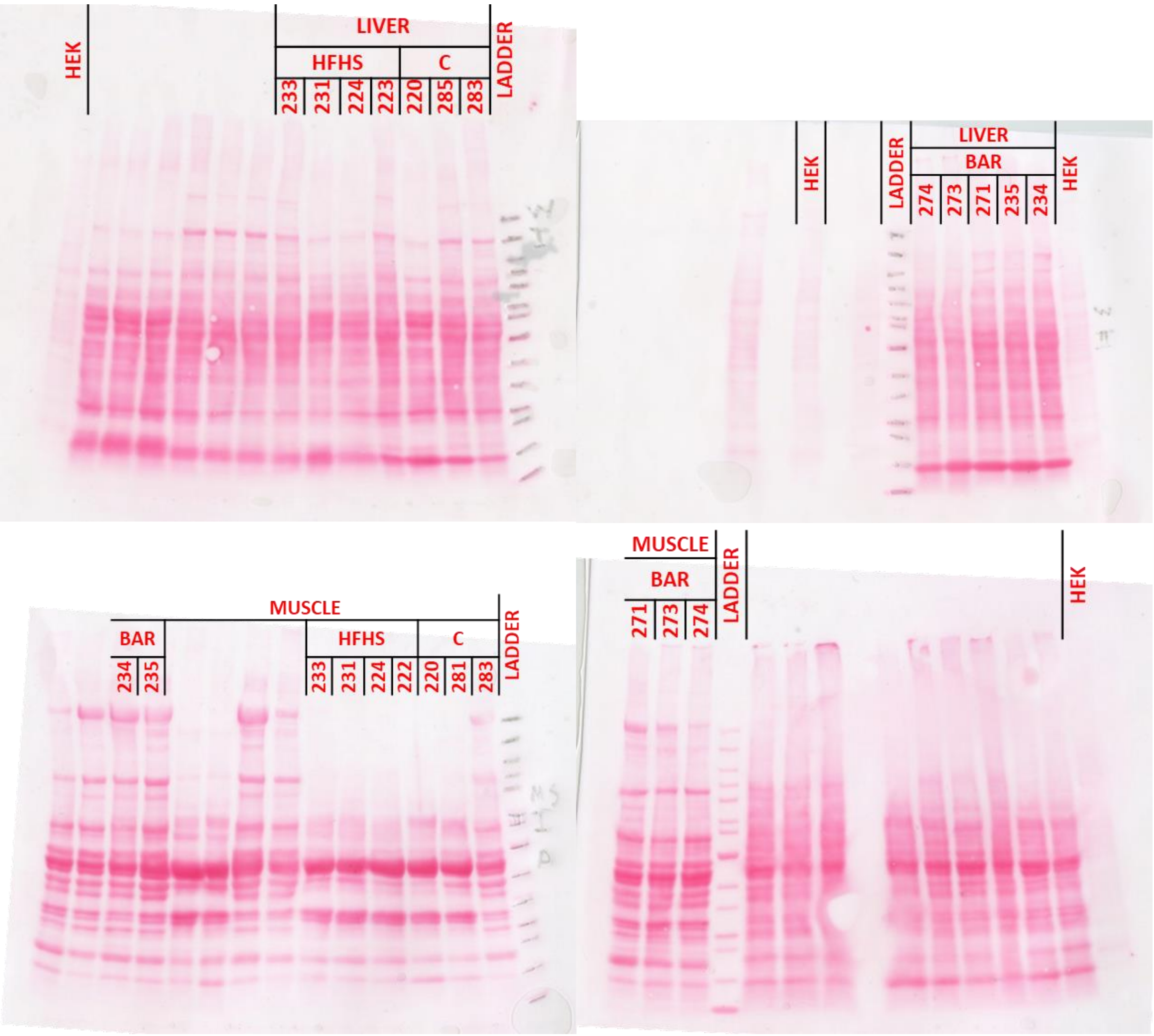

Suplementary Figure 6. **Western blot analysis of ALKBH4 protein.** The bars selected for analysis are marked with red squares. The overexposed signal is highlighted in red. The amount of signal from the sample was normalised by dividing the value by the amount of signal from the HEK293 cell line (HEK) sample or the signal from the ladder band. Invastigated groups: C - Standard diet (n = 6); HFHS - high energy diet with developed obesity and insulin resistance (n = 4); BAR - Pigs after bariatric surgery (n=6); Invastigated tissues: M – muscle; P – pancreas; L – Liver; F – Adipose tissue. I – Image of exposition from which densitometric data were obtained; II – Overview image of long exposition showing the blots including their framing, possible cutting locations and molecular ladder markings. III – Overview image of the blots after Ponceu Red stayning, for total protein verification after protein transfer on the membrane.

I

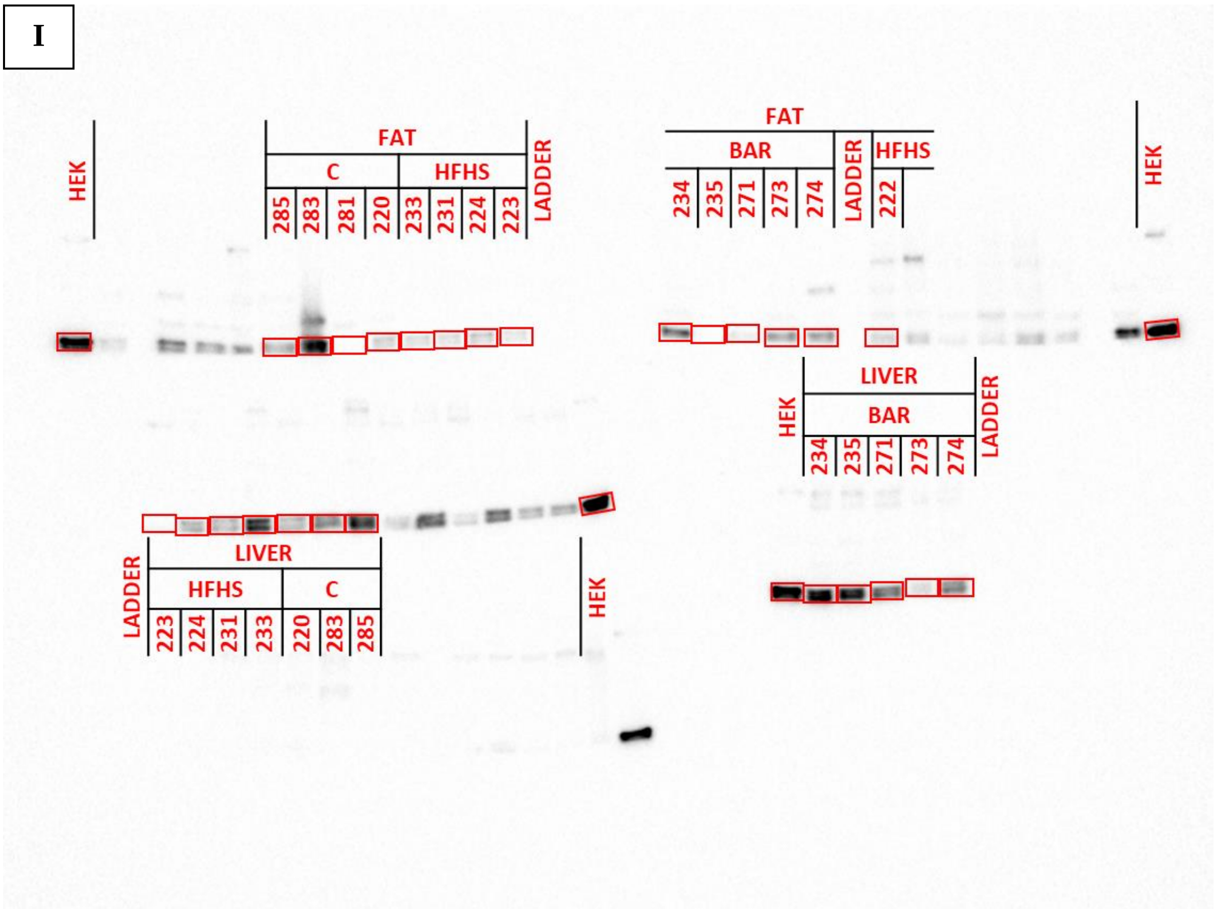
HEK
FAT
C
HFHS
285
283
281
220
233
231
224
223
LADDER
FAT
BAR
LADDER
HFHS
234
235
271
273
274
222
HEK
LIVER
BAR
HEK
234
235
271
273
274
LADDER
LADDER
LIVER
HFHS
C
223
224
231
233
220
283
285
HEK

II

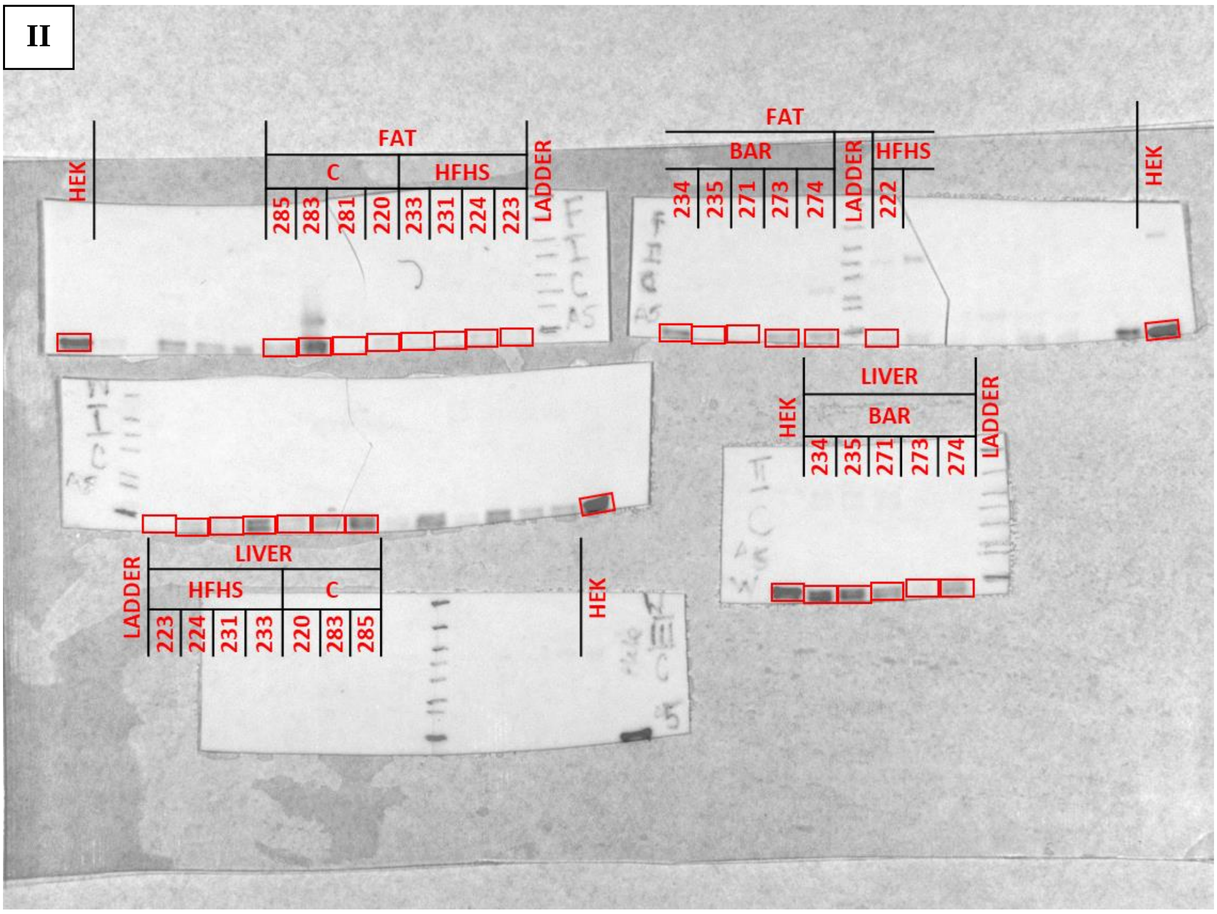
HEK
FAT
C
HFHS
285
283
281
220
233
231
224
223
LADDER
FAT
BAR
LADDER
HFHS
234
235
271
273
274
222
HEK
LIVER
BAR
HEK
234
235
271
273
274
LADDER
LADDER
LIVER
HFHS
C
223
224
231
233
220
283
285
HEK

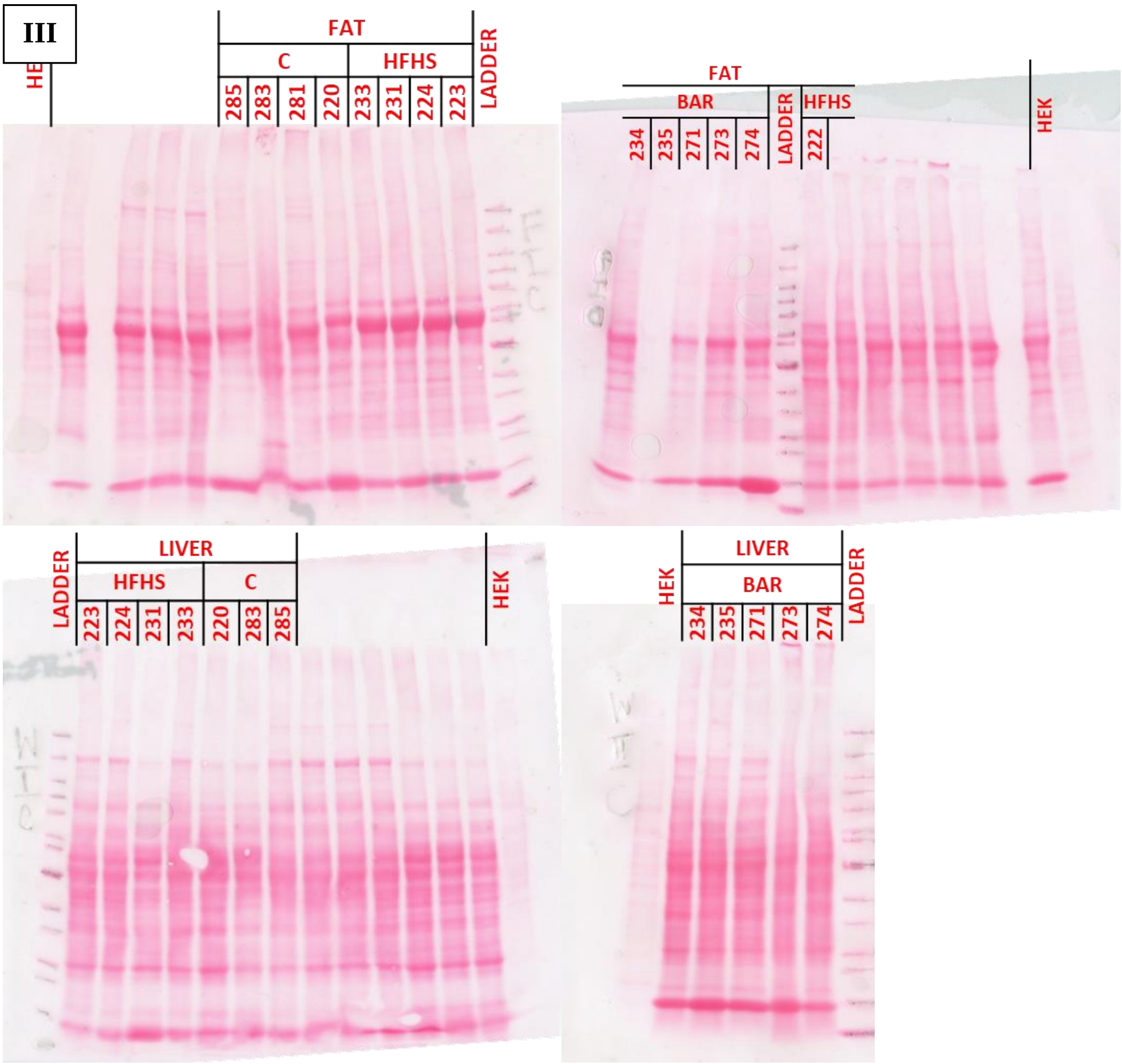

Suplementary Figure 7. **Western blot analysis of ALKBH5 protein.** The bars selected for analysis are marked with red squares. The overexposed signal is highlighted in red. The amount of signal from the sample was normalised by dividing the value by the amount of signal from the HEK293 cell line (HEK) sample. Invastigated groups: C - Standard diet (n = 6); HFHS - high energy diet with developed obesity and insulin resistance (n = 4); BAR - Pigs after bariatric surgery (n=6); Invastigated tissues: M – muscle; P – pancreas; L – Liver; F – Adipose tissue. I – Image of exposition from which densitometric data were obtained; II – Overview image of long exposition showing the blots including their framing, possible cutting locations and molecular ladder markings. III – Overview image of the blots after Ponceu Red stayning, for total protein verification after protein transfer on the membrane.

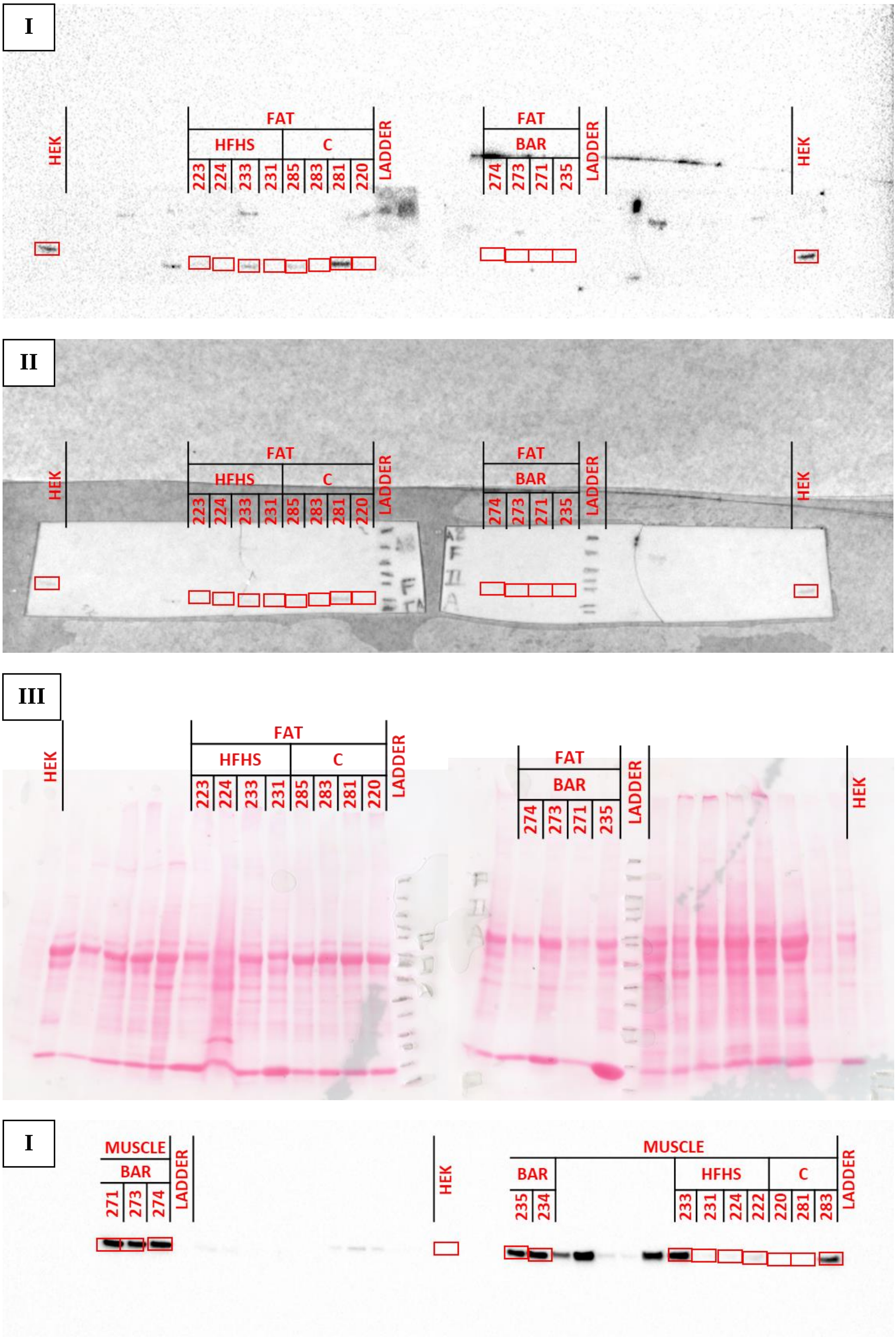
I
HEK
FAT
HFHS
C
223 224 233 231 285 283 281 220
LADDER
FAT
BAR
274 273 271 235
LADDER
HEK
II
HEK
FAT
HFHS
C
223 224 233 231 285 283 281 220
LADDER
FAT
BAR
274 273 271 235
LADDER
HEK
III
HEK
FAT
HFHS
C
223 224 233 231 285 283 281 220
LADDER
FAT
BAR
274 273 271 235
LADDER
HEK
I
MUSCLE
BAR
271 273 274
LADDER
HEK
MUSCLE
BAR
235 234
HFHS
233 231 224 222
C
220 281 283
LADDER

II

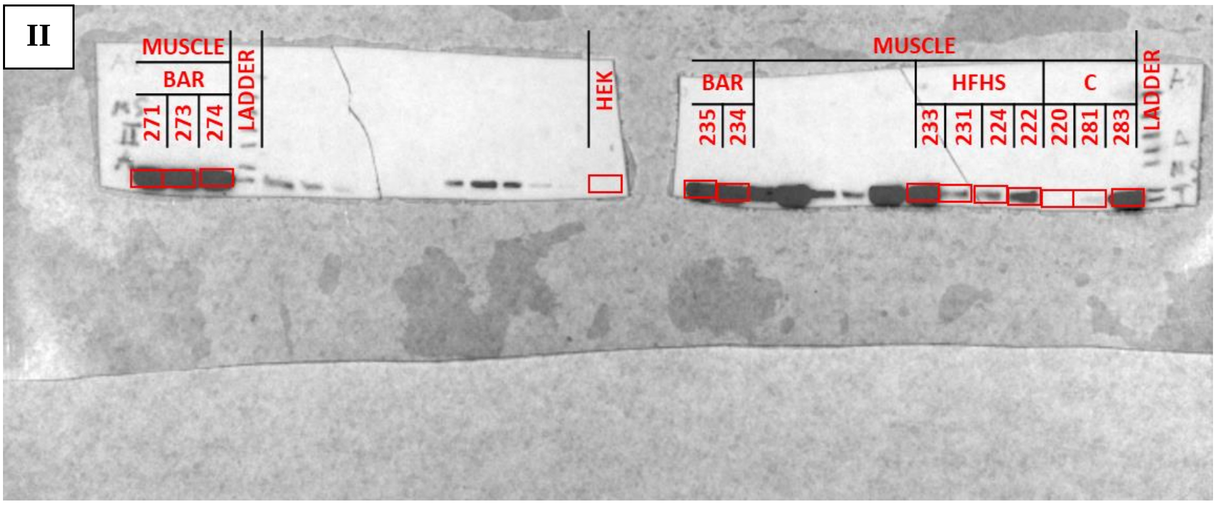
MUSCLE
BAR
271
273
274
LADDER
HEK
MUSCLE
BAR
235
234
HFHS
233
231
224
222
C
220
281
283
LADDER

III

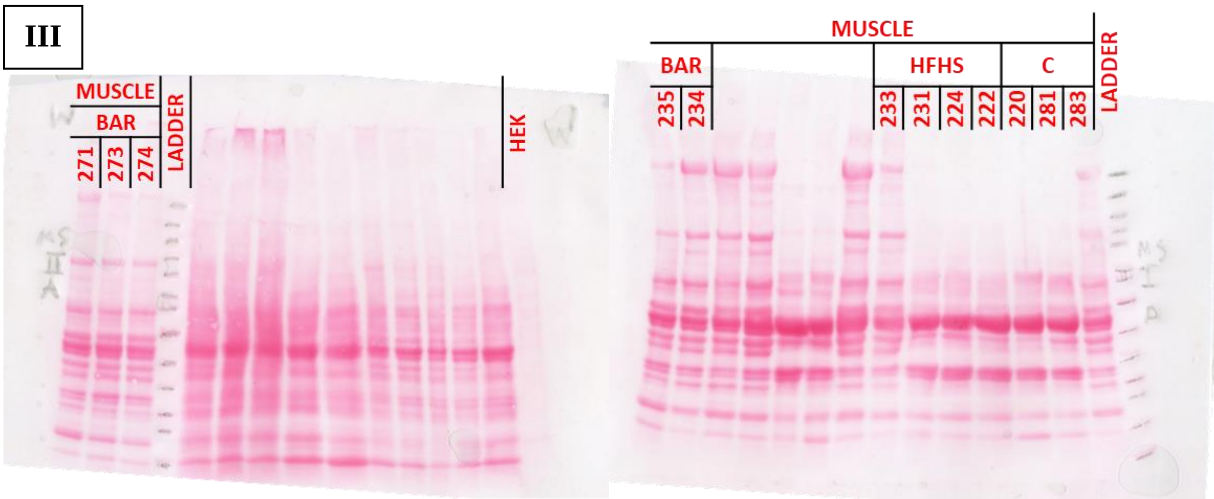
MUSCLE
BAR
271
273
274
LADDER
HEK
MUSCLE
BAR
235
234
HFHS
233
231
224
222
C
220
281
283
LADDER

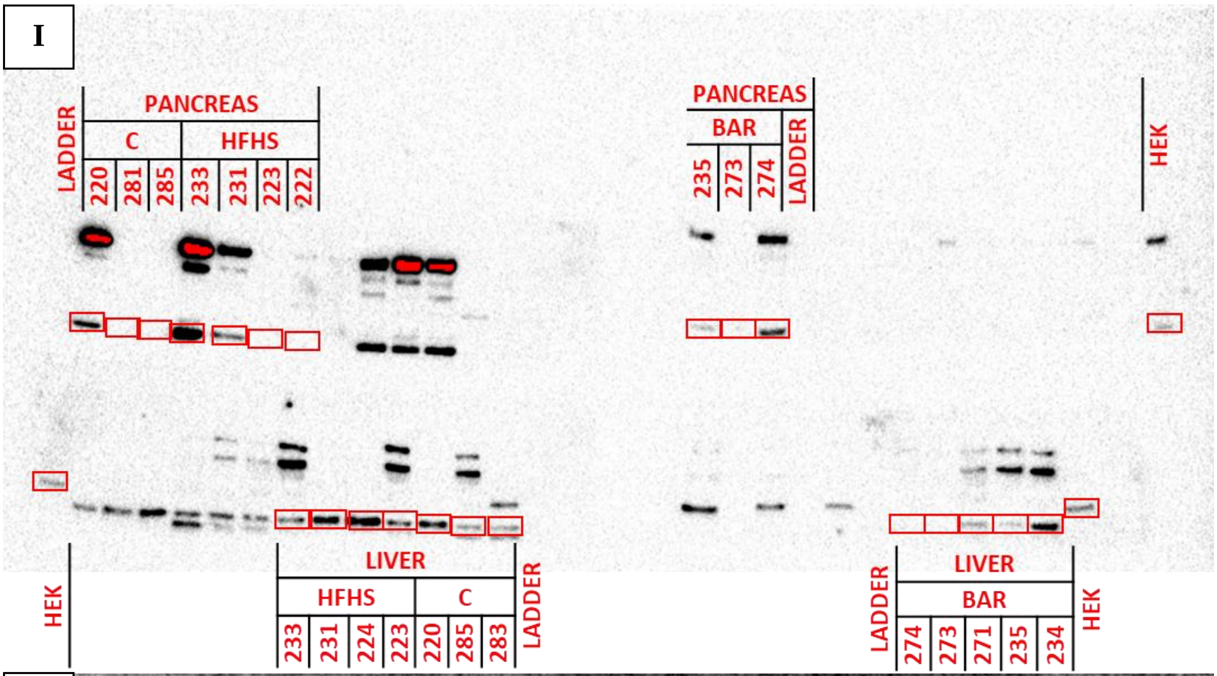
I
PANCREAS
C
HFHS
LADDER
220
281
285
233
231
223
222
PANCREAS
BAR
235
273
274
LADDER
HEK
HEK
LIVER
HFHS
C
233
231
224
223
220
285
283
LADDER
LADDER
LIVER
BAR
274
273
271
235
234
HEK

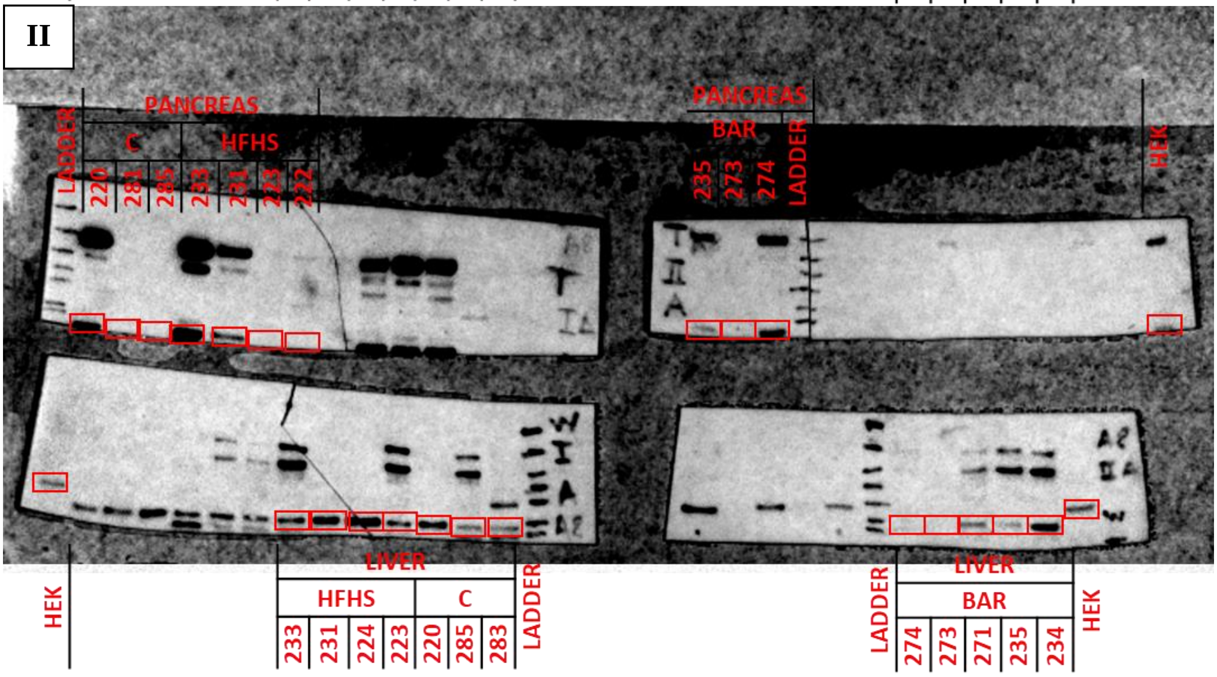
II
PANCREAS
C
HFHS
LADDER
220
281
285
233
231
223
222
PANCREAS
BAR
235
273
274
LADDER
HEK
HEK
LIVER
HFHS
C
233
231
224
223
220
285
283
LADDER
LADDER
LIVER
BAR
274
273
271
235
234
HEK

III

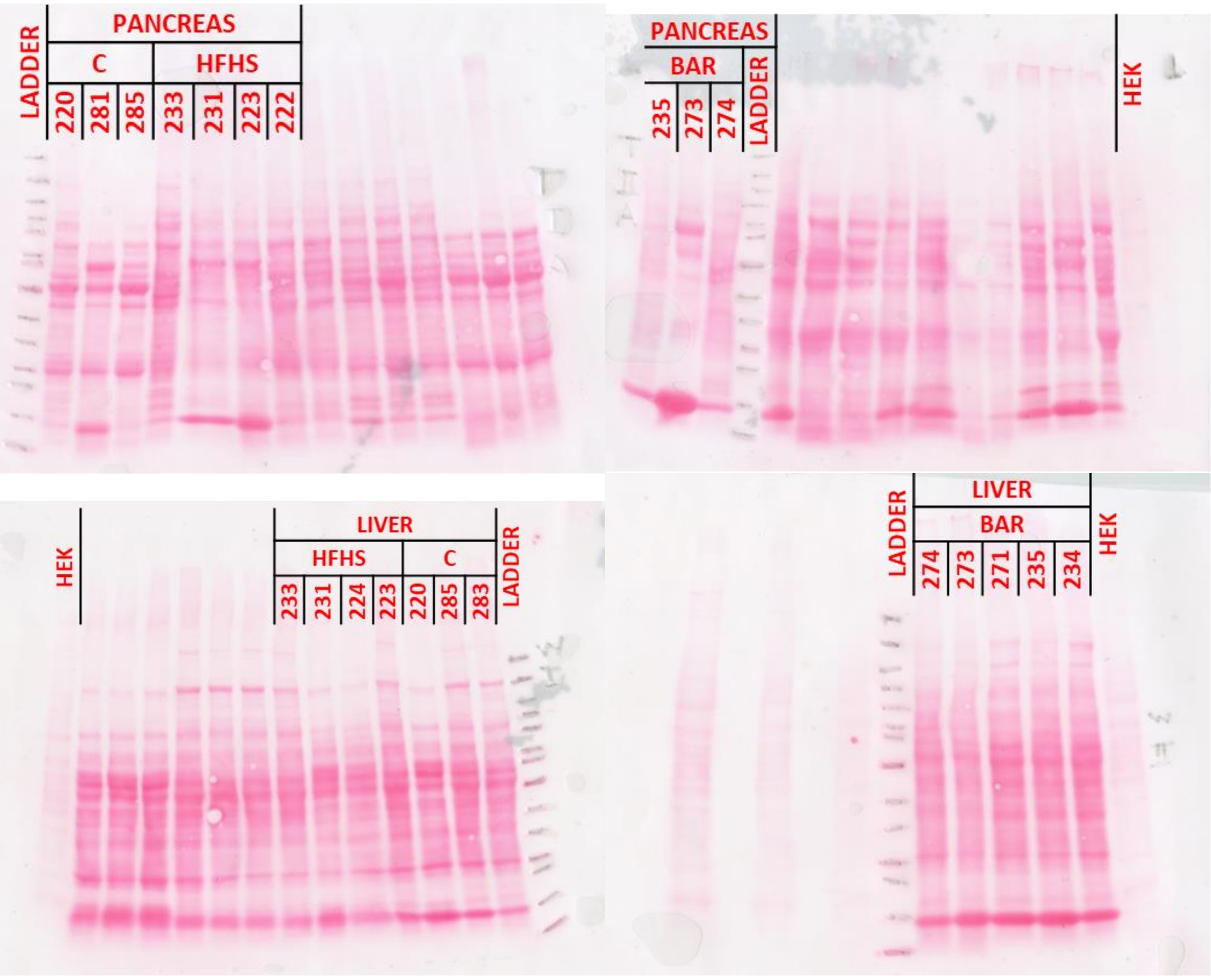

Suplementary Figure 7. **Western blot analysis of ALKBH8 protein.** The bars selected for analysis are marked with red squares. The overexposed signal is highlighted in red. The amount of signal from the sample was normalised by dividing the value by the amount of signal from the HEK293 cell line (HEK) sample. Invastigated groups: C - Standard diet (n = 6); HFHS - high energy diet with developed obesity and insulin resistance (n = 4); BAR - Pigs after bariatric surgery (n=6); Invastigated tissues: M – muscle; P – pancreas; L – Liver; F – Adipose tissue. I – Image of exposition from which densitometric data were obtained; II – Overview image of long exposition showing the blots including their framing, possible cutting locations and molecular ladder markings. III – Overview image of the blots after Ponceu Red stayning, for total protein verification after protein transfer on the membrane.
